# Understanding demographic events and migration patterns in two urban slums of Nairobi City in Kenya

**DOI:** 10.1038/s41598-024-79895-x

**Published:** 2024-11-21

**Authors:** Evans Omondi, Samuel Iddi, Sharon Chepkemoi, Bylhah Mugotitsa, Steve Cygu, Boscow Okumu, Abdhalah Ziraba, Damazo T. Kadengye, Agnes Kiragga

**Affiliations:** 1 African Population and Health Research Center (APHRC), Nairobi, Kenya; 2 Institute of Mathematical Sciences, Strathmore University, Nairobi, Kenya; 3 Department of Statistics and Actuarial Science, University of Ghana, Legon-Accra, Ghana; 4 Strathmore University Business School, Strathmore University, Nairobi, Kenya; 5 Environment for Development Initiative, Department of Economics, University of Nairobi, Nairobi, Kenya

**Keywords:** Population, Demography, Urban slums, Migration, Surveillance, Multi-state model, Environmental social sciences, Mathematics and computing

## Abstract

Understanding the dynamics of movements between different demographic events is essential for informing effective population management strategies. This study aims to characterize the trajectories of demographic and other vital events within the Nairobi Urban Health and Demographic Surveillance System (NUHDSS). Thus, it intends to unravel patterns and trends that can guide the development of targeted policies and interventions to address the population’s evolving needs. Using a continuous-time homogeneous multi-state Markov model, longitudinal data from 223,350 individuals in Korogocho and Viwandani urban slums, we study the enumeration, births, deaths, and migrations among urban poor in Nairobi, shedding light on population dynamics and movements over time, disaggregated by gender. Findings indicate a positive net migration in population per thousand in 2002, dropping in 2004, with Viwandani consistently showing higher birth rates than Korogocho. Males generally have higher death rates than females. Females from Viwandani are 39.0% more likely to exit after enumeration compared to Korogocho, while males are 35.6% more likely to move from enumeration to exit compared to males from Korogocho. Both genders from Viwandani have a decreased likelihood of moving from birth to death compared to Korogocho. Our findings provide unique insights into migration in urban Kenya, the frequency and movement to different demographic events and any gender differences that warrant strategic policies for effective population and health planning in Africa. These findings can inform the design of effective health interventions that are often affected by migration and population growth.

## Introduction

Urban slum areas are densely populated within cities and are characterized by inadequate housing, poor infrastructure, and limited access to basic services such as clean water, sanitation and healthcare^1–4^. Typically, these areas emerge as informal settlements due to rapid urbanization, where the demand for affordable housing appears to be higher than the supply consequently leading to the development of unregulated and poorly serviced neighborhoods^4–6^. As a result, residents of urban slums face significant socio-economic challenges, including high levels of poverty, unemployment, and vulnerability to health risks due to overcrowded living conditions^6,7^. Within Nairobi City, Korogocho and Viwandani are two prominent examples of such urban slums, each with its unique socio-economic characteristics, infrastructure limitations, and demographic profiles that influence the daily lives of their residents^6,8^.

Korogocho informal settlements has an estimated population of over 150,000 people living within 1.5 square kilometers^9^. The area is marked by high levels of poverty, with most residents surviving on less than $2 per day, primarily through informal employment such as casual labor and small-scale trading^6,8^. Infrastructure in Korogocho is underdeveloped and housing is often made from makeshift materials like corrugated iron sheets^9^. Additionally, access to essential services such as clean water, sanitation, and electricity is limited and unreliable^8,10^. The demographic profile of Korogocho is predominantly young, with a diverse ethnic composition including Kikuyu, Luo, Luhya, and Kamba communities amongst others^6^. Many residents are migrants from rural areas, drawn to Nairobi by the hope of better economic opportunities^11^. On the other hand, Viwandani informal settlement boasts of estimated population of over 100,000 with many residents engaged in low-wage industrial work or informal sector jobs, such as casual labor, petty trading, and small-scale manufacturing^9^. The economic conditions are challenging, with many households living below the poverty line and relying on unstable sources of income. The infrastructure is underdeveloped, similar to Korogocho. Access to essential services such as water, sanitation, and electricity is limited, with frequent interruptions^10^. Demographically, Viwandani has a youthful population, with a significant proportion of residents under the age of 30. The settlement is also ethnically diverse, drawing people from various ethnic backgrounds across Kenya, including Kikuyu, Luo, Luhya, and Kamba^6^. Similar to Korogocho, many residents in Viwandani are migrants from rural areas who have moved to Nairobi in search of employment opportunities in the nearby industrial zones^11^.

In urban slums, there exists various dynamics of urbanization and population change which can be anchored within the frameworks of Demographic Transition Theory and Urban Transition Theory^12^. Demographic Transition Theory provides insights into the stages of population change, particularly the shifts in birth and death rates as societies undergo transition from pre-industrial to industrialized economies^12–14^. This theory is essential for understanding the demographic shifts observed in the two Nairobi slums, where rapid urbanization and economic pressures are accelerating changes in both fertility and mortality patterns. Additionally, Urban Transition Theory is pertinent in explaining the transition from rural to urban living that is marked by shifts in population distribution, economic activities and social structures^15^. This theoretical approach allows for the description of changes occurring within these two informal settlements and also to link them to the broader urbanization processes affecting Nairobi city. Korogocho and Viwandani informal settlements are characterized by various demographic events which are pivotal in shaping demographic dynamics and population movement, serving as fundamental indicators of population growth and mortality rates^16^. Birth and death events, in particular, provide essential insights into population trends and mortality patterns. Additionally, in and out-migration, significantly influence population composition and distribution within the given demographic areas, and are driven by economic, social, and environmental factors^17^. Internal change of place of residence or broadly described as entry and exit, representing movements into and out of defined administrative areas or systems such as households and communities, offer valuable information about demographic transitions^12,18^.

The complexity of migration phenomena has garnered interdisciplinary research attention, reflecting the features of migration dynamics^19^. Migration is not only a subject of academic interest but also a crucial consideration for governments and policymakers. Effective migration management and urbanization policies are essential for addressing the challenges posed by population mobility and ensuring sustainable development. Migration of individuals from one geographical setting to another is driven by complex interaction between pull and push factors^20^. Push factors are those that compel individuals to migrate due to adverse conditions such as economic hardship, political instability, social inequality, and environmental disasters. These factors include food insecurity, limited access to clean water, unemployment, poverty, climate change impacts, conflict, and war^21^. On the other hand, pull factors are those that drive migration into new destinations by offering better prospects and conditions. They range from economic opportunities such as higher wages and job opportunities, political stability, religious tolerance and freedom from persecution, access to quality education and healthcare, and good or improved living conditions^22^. Furthermore, social networks such as marriages, family ties and friends who have already migrated and provide information about their destinations, further influence migration trends^23^. Thus, understanding the diverse factors influencing migration patterns is essential for designing informed policies and interventions to support migrant populations and foster inclusive urban development.

Despite the significant socio-economic challenges faced by residents of Korogocho and Viwandani, the dynamics of population movements, births, and deaths remain scantly understood, particularly in the context of rapid urbanization^24^. These demographic events are pivotal in shaping the overall population structure and directly impacting the health and well-being of the residents in these areas^25^. However, capturing these dynamics requires a robust analytical framework that can capture the transitions between various demographic states and quantify the impact of socio-demographic factors on these transitions. In this study, we propose a multi-state modelling framework to examine the transitions between different demographic events in the two Nairobi urban slums of Korogocho and Viwandani. We also examine the association between key socio-demographic factors, such as age and ethnicity these demographic events. More precisely, our aim is to investigate the changes in demographic events within the Demographic Surveillance Systems (DSS) and interpret these changes in the context of population dynamics and urbanization trends. The model quantifies movement through demographic states and estimates the duration of stay in each state. This approach is capable of offering insights for policymakers aimed at improving living conditions in urban slums.

## Methods

### Data source, and variables

To understand population dynamics in countries with weak civil registration and vital statistics systems, Health and Demographic Surveillance Systems (HDSSs) have been established in some countries, both in rural and urban areas including informal urban settlements. The Nairobi Urban and Demographic Surveillance System (NUHDSS) is one such system that provides information on the initiation and termination of residency in slum areas, offering insights into population mobility, births, deaths, living arrangement and urbanization trends^6^. The NUHDSS was established during the era of the Millennium Development Goals (MDGs) and pioneered by African Population and Health Research Center (APHRC) in consultation with Nairobi City Council and community members of two slums, Korogocho and Viwandani in August 2002, with the main goal of generating information for decision-making specific to the slum residents of Nairobi^6^. It is a longitudinal observational study conducted in the two urban slum settlements. This study uses residency data from the NUHDSS, which documents the duration of an individual’s stay in a specific geographical area. The data collection process involved administering a series of questionnaires to the entire population or residents. In the NUHDSS, a resident is defined as a person who has lived within the DSA for a minimum period of more than 180 days. Every household in the slum areas was visited, and the status of each member or respondent was updated. A respondent or member of the household refers to the person who is expected to respond to a set of questions or a questionnaire. Each questionnaire specifies who is eligible to be a respondent which is defined as any capable adult member of a given household. In situations where no household members are available for the interview, one may interview credible adult neighbors. In such instances one is required to indicate that on the survey form. The NUHDSS was updated through rounds of individual and household status surveys, capturing data on fertility, mortality, migration, marital status, educational attainment, ethnicity, household composition, selected child health indicators, and household socio-economic status. Status updates were carried out every six months.

**Fig. 1:**
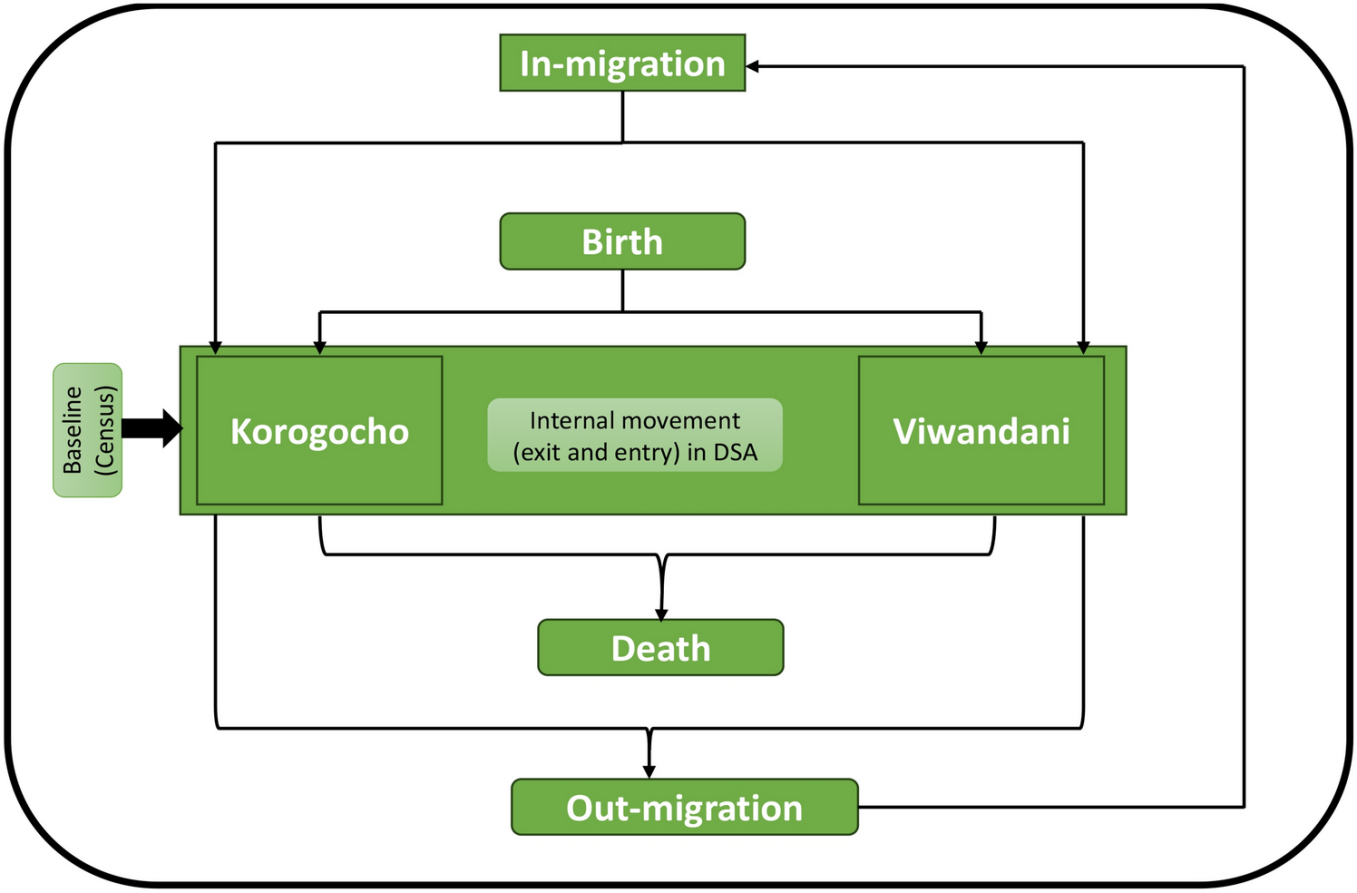
The flowchart showing the population events updated through period enumeration in any Demographic Surveillance Area (DSA).

This dataset offers insightful information into residency initiation in these areas, encompassing key demographic events such as birth, in-migration, out-migration, internal changes of residence (entry vs. exit), and death, as illustrated in Fig. 1.

Our outcome variable of interest is the demographic event, split into seven categories: enumeration, birth, exit, entry, out-migration, in-migration, and death. The categories of this variable are described below:

- *Enumeration* This refers to the systematic process of counting and recording the individuals and households within the Demographic Surveillance Areas (DSA). In this case, the enumeration event occurred once in the year 2002 within the demographic surveillance area. This event was used to establish the baseline population for subsequent surveillance and research hence crucial for tracking demographic changes, health trends, and other population dynamics over time.
- *Birth* A birth refers an individual born within the Demographic Surveillance Area by a resident of of the DSA.
- *Exit and Entry* These refer to a situation where a resident has moved out a household and acquired a new one within the DSA. Thus, these movements as described as the internal movement within the DSA.
- In-migration - This refers to the movement of an individual residing outside the DSA into into the DSA. Such an individual is regarded an in-migrant if the individual moves in to the DSA and resides permanently for more than 180 days.
- Out-migration - This refers to the movement of an individual out of the DSA and stayed away from the area for an extend period of more than 180 days.
- Death - This refers to the death of resident within the DSA or outside the DSA but before qualifying to be an out-migrant. The death event is a permanent end event.

All these events were updated based residency and membership statuses which were assigned at the start of the DSS. Additionally other social demographic variables captured include gender, ethnicity, place of birth, age, and living condition, that is, residing in the slum area. The analysis in this study is based on 223,350 individuals captured in Demographic Surveillance Area through different rounds of status updates.

#### Ethics

The NUHDSS study was performed in accordance with the declaration of Helsinki and received ethical clearance from the Kenya Medical Research Institute (KEMRI) in 2002 (Reference: NON SSC 271A) which has consequently been renewed over the years. Informed consent was obtained from all participants. The analysis was based on an anonymized dataset with no identifiable information on the study participants.

### Statistical methodology

**Fig. 2:**
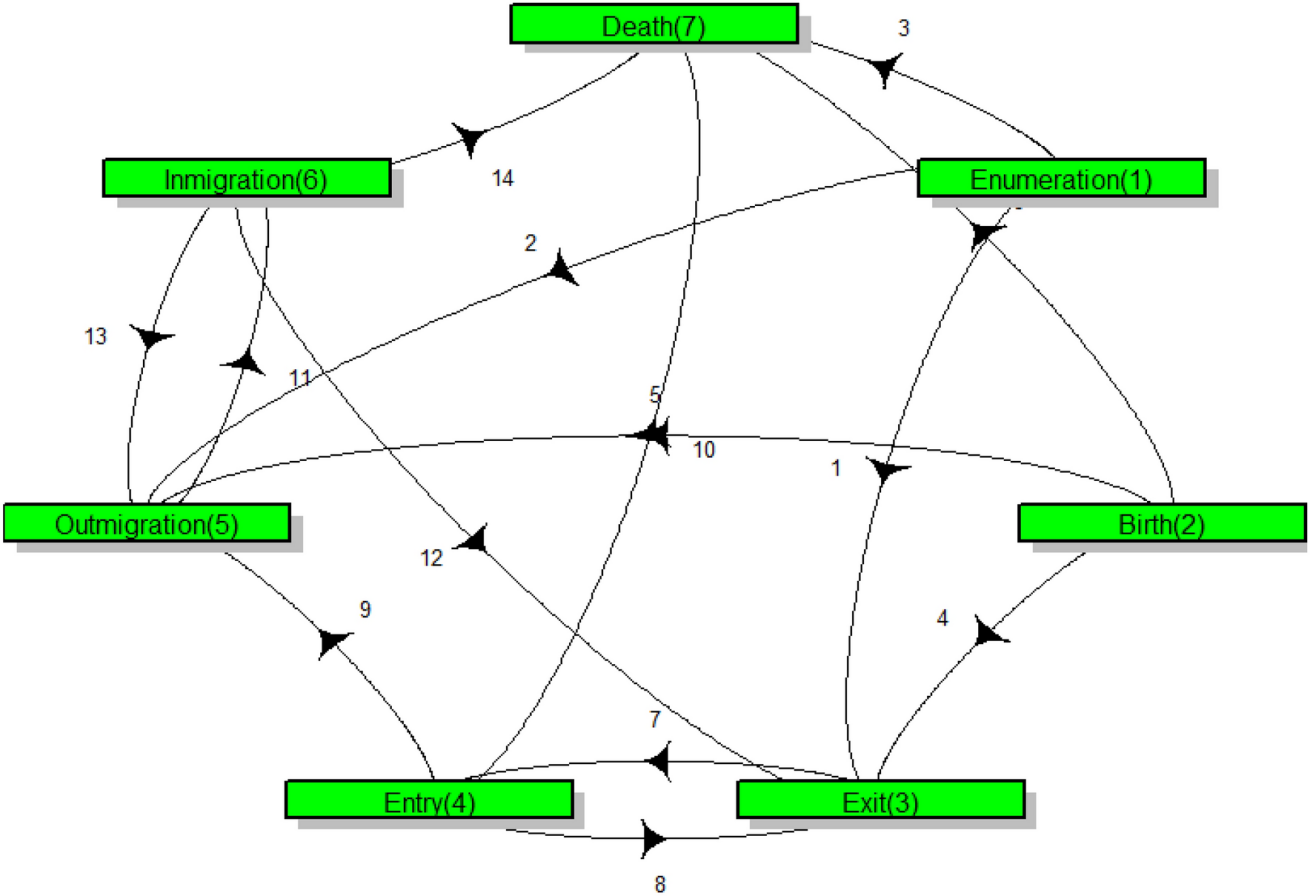
The flowchart showing the multi-state model describing the transition from one residence event to the next. Each arrow corresponds to a possible transition $(n = 14)$. For the reasons of modelling and ease of interpretation the seven residency states are named as follows: State 1: Enumeration, State 2: Birth, State 3: Exit, State 4: Entry, State 5: Out-migration, State 6: In-migration and State 7: Death.

Multi-state models (MSMs) characterize the movement of individuals through successive states of an event. For example, MSMs have been utilized to elucidate the occurrence and progression of patients undergoing treatments, such as cancer and other diseases^26,27^. Moreover, MSMs have been applied to examine socioeconomic disparities in life expectancy, accounting for multi-morbidity^28^. Multi-state modeling assumes that individuals are observed in one of a finite number of states at any given time during the observation period, making it suitable for processes involving transitions between well-defined states^29^. Thus, residency events can be conceptualized as individuals transitioning through various states, with each event representing a distinct profile. MSMs expand upon traditional survival models by accommodating more than two states, providing a robust probabilistic framework for analyzing complex longitudinal event history data and generating predictive insights. The versatility of MSMs renders them invaluable for analyzing residency event data and comprehending the intricate dynamics of population mobility and urbanization trends. However, despite their considerable potential, the application of MSMs to understanding of population dynamics remains limited, particularly in the African context. Additionally, comprehensive studies investigating the influence of socio-demographic characteristics such as age, gender, and lifestyle factors on transitions between demographic events are scarce, yet important for urban planning. Multi-state modelling was used to analyze the complex dynamic of the demographic events over time. Multi-state model used in this study is suitable since it is able to account for the duration individuals spend in each state and handle transitions between multiple states over time. This modeling framework is employed based on two key advantages: first, the model analysis incorporates person-years at risk, as it estimates transition rates based on the time individuals spend in each state before transitioning to another. This approach effectively accounts for the duration each individual is at risk of experiencing the event. Second, the model accommodates right-censoring, which is essential given the significant movement in and out of the NUHDSS. The model assumes that censored observations are non-informative and adjusts the transition rates accordingly to provide unbiased estimates. To keep a smooth functioning of the model, a seven-state continuous time homogeneous multi-state model was used. The model analysis was stratified by gender of the individuals. The adopted multi-state model as shown in Fig. 2 analyses how individuals move between different states (demographic events) over time. The arrows indicate the instantaneous state-to-state transitions which are bi-directional and reflects the dynamic nature of the individuals moving in and out of the different demographic events (states) in the NUHDSS over time.

Let *X*(*t*) represent the state occupied by an individual at time *t* taking on the values $1,2, \ldots , \{7\}$ and let $H(t)=\left\{ X(s), ~ 0\le s\le t\right\}$ be the history of occupancy up to age $t, ~ t>0$. The time variable, *t*, is measured in days. In addition, consider a set of fixed covariates in vector $\textbf{z}$. Thus, the risk of movement between states *i* and *j* can be described by the transition intensities given by,

1 $$\begin{aligned} \left. \begin{aligned} \lambda _{ij}(t|H(t^{-}), \textbf{z}) = \lim _{\Delta t\rightarrow 0} \frac{P\left( X(t+\Delta t) = j|X(t) = i, H(t^{-}, \textbf{z})\right) }{\Delta t}, \end{aligned}\right. \end{aligned}$$

for $j\ne i, ~~ i,j =1, \ldots , \{7\}$. Here, the transition intensities are assumed to depend only on the state currently occupied. Thus, we adopt time-homogeneous Markov model given by

2 $$\begin{aligned} \left. \begin{aligned} \lambda _{ij}(t|H(t^{-}), \textbf{z}) = \lambda _{ij}(t|\textbf{z}). \end{aligned}\right. \end{aligned}$$

To account for the covariate effects (slum area, age, gender, ethnicity, and area of birth ) on the transition intensities, the instantaneous transition rates is expressed as the proportional hazard model given by

3 $$\begin{aligned} \left. \begin{aligned} \lambda _{ij}(t|H(t^{-}), \textbf{z}) = \lambda _{ij}(t|\textbf{z})(t|H(t^{-}))\exp \{\mathbf {z'}\mathbf {\beta }_{ij}\}. \end{aligned}\right. \end{aligned}$$

The parameter vector $\mathbf {\beta }_{ij}$ is specific to each $i\rightarrow j$ transition, i.e., $\mathbf {\beta }_{ij}$ represents the effect of each covariate on the hazard of moving from state *i* to state *j*. In this model, each state represents a specific status an individual can be in, and the model estimates the probabilities of moving between these states based on factors such as age, ethnicity, and other covariates. Additionally, it accounts for time-dependent changes which allows for predictions of how transitions might evolve in the future under different conditions. The mathematical formulations in Eqs.(1), (2) and (3) assumes that these transitions only depend on the current state and the covariates. Additionally, the model uses a proportional hazards approach, where the effect of covariates on the transition rate is captured using an exponential function. This structure allows us to estimate the likelihood of individuals changing states while accounting for important demographic and socio-economic factors. Hazards regression analysis was used to estimate the hazard ratios (HRs) and 95% confidence intervals (CIs) for demographic events. These analyses were conducted in **R** (version 4.4.1, R Core Team) and R Studio (version 2024.04.0+735, R Studio Team) software, using the *msm* package^30,31^.

## Results

### Demographic indicators

We began our analysis by conducting exploratory statistical analysis such as of summary measures of the population within the DSA and the core demographic events. Percentages, frequencies, and rate per 1000 individuals summaries are utilized for categorical and continuous factors, respectively. In Table 1 we present results about the non-changing variables such as gender and place of birth as they provide essential demographic context of the population within the Demographic Surveillance Areas. A total of 223,350 individuals were included under the surveillance system in the NUHDSS between 2002 and 2015. The highest proportion reside in Viwandani (61.0%), 45.3% were female, and majority were of the Kamba and Kikuyu ethnic groups, 26.8% and 26.2%, respectively. Viwandani area is also predominantly inhabited by Kamba (39.2%) while Korogocho slums were found to be predominantly inhabited by Kikuyus (30.8%) and Luos (28.1%). Overall, the leading communities in the two slums were Kambas (26.8%) followed by Kikuyus (26.2%). Other significant ethnic groups include Luhya (16.7%), Luo (14.9%), and a category labeled ‘Other’(15.4%). Notably, 15.5% of the residents were born within the DSA, while 9.0% originated from other slums within Nairobi. The findings also reveal that 68.9% of the overall slum dwellers were born in rural areas. In addition, the median age over the span of 2002 to 2015 consistently remained between 21 and 23 years with the lower quartile being 10 years and the upper quartile being 34 years as shown in Supplementary Table S1.

**Table 1:** Social and demographic variables of survey participants in the Demographic Surveillance Area (%).

Characteristic	Slum area		Gender		
	Korogocho 87,213 (39.0)	Viwandani 136,137 (61.0)	Female 101,187 (45.3)	Male 122,163 (54.7)	Overall 223,350
Ethnicity					
Kamba	7.6	39.2	25.2	28.2	26.8
Kikuyu	30.8	23.2	27.0	25.5	26.3
Luhya	21.6	13.6	17.3	16.3	16.7
Luo	28.1	6.5	15.9	14.1	14.9
Other	11.9	17.6	14.6	16.0	15.4
Area of birth					
Rural Kenya	56.8	76.7	68.1	69.7	68.9
Within same DSA slum	21.6	11.5	15.9	15.1	15.5
Nairobi non-slum	13.4	6.2	9.4	8.7	9.0
Other places	8.1	5.6	6.7	6.6	6.6

### Population dynamics

**Fig. 3:**
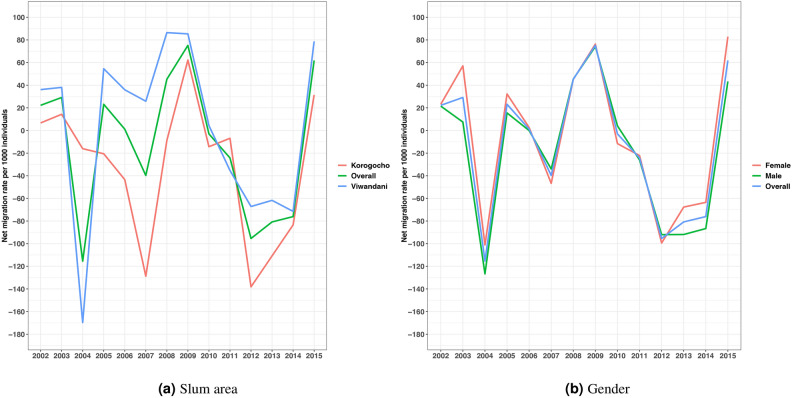
The net migration rates per 1000 individuals in the Nairobi Urban Demographic and Health Surveillance Site from 2002 to 2015.

We examine the demographic events that characterize the population changes, that is, migration patterns, birth and death rates, and urbanization trends in the DSA. The findings in Fig. 3a illustrate the trends in net migration rates per 1000 individuals from 2002 to 2015 for Korogocho, Viwandani, and the overall population. Both Korogocho and Viwandani show consistently negative net migration rates, indicating persistent out-migration, with more people leaving than coming into the areas. Korogocho experiences a particularly steep decline around 2006, suggesting a notable increase in outmigration during that period. Viwandani shows less drastic fluctuations but similarly remains in negative territory. The overall trend combines these patterns, reflecting a general outflow from the two slum areas throughout the study period. This could suggest significant socio-economic challenges driving population loss in these communities. Additionally, the findings in Fig. 3b show that both male and female migration rates exhibit predominantly negative trends, indicating high outmigration tendencies in the areas. Males generally show more pronounced negative migration rates, especially between 2006 and 2008. Females follow a similar trend, but with slightly less negative rates. The combined overall trend reflects these patterns, confirming sustained net outmigration across both genders throughout the study period. This consistent outmigration may point to shared socio-economic or environmental challenges affecting both males and females in the area. More results about the patterns of migration are given in Supplementary Table S2.

**Fig. 4:**
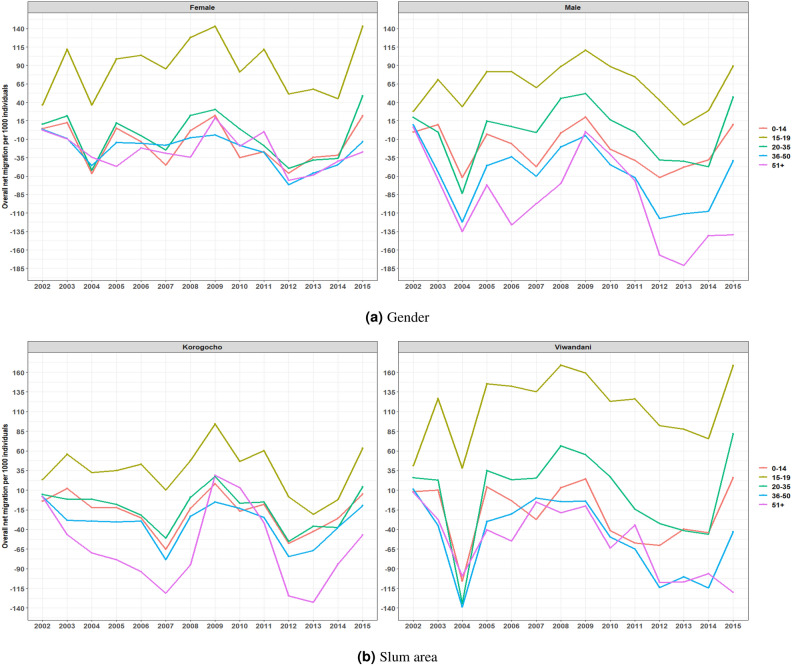
The age group specific overall net migration rates by gender and slum area.

In Fig. 4a we show the overall net migration rates per 1000 individuals for different age groups, separated by gender (female on the left panel and male on the right panel). The various lines represent distinct age categories from 0–14, 15–19, 20–35, 36–50, and 51+ years. The trend in Fig. 4a indicates fluctuations in migration patterns over time, with notable variations among different age groups. Those aged 15–19 years consistently exhibit the highest net migration rates, showing positive values, suggesting higher in-migration and lower out-migration compared to other groups. On the other hand, those in the age groups 36–50 and 51+ years tend to show more negative net migration rates, indicating higher out-migration. There is a sharp decline around 2003–2004 for most age groups. The patterns remain somewhat consistent across both genders, though the male group has slightly more pronounced negative net migration rates in certain years (e.g., 2009–2013). This reflects how migration behavior is influenced by age and gender dynamics in this population over time.

Additionally, in Fig. 4b we depict overall net migration rates per 1000 individuals for different age groups across two informal settlements in Nairobi: Korogocho and Viwandani. The trends highlight significant differences between the age groups and between the two settlements. In both Korogocho and Viwandani, the 15–19 age group shows consistently higher positive net migration rates, indicating that more people in this age group are moving into these areas than leaving. In contrast, older age groups (especially 36–50 and 51+) generally show negative migration rates, implying that more individuals in these age groups are moving out of the settlements. Viwandani shows more volatility in migration trends, particularly in the 2004–2009 period, where there are sharp declines followed by gradual recoveries across most age groups. Furthermore, Korogocho also has fluctuations, but they are less pronounced than those in Viwandani. Both settlements experience an overall increase in net migration for the 15–19 and 20–35 age groups towards the end of the period. These findings suggest that younger people are increasingly moving into these informal settlements, while older age groups are more to out-migrate.

**Fig. 5:**
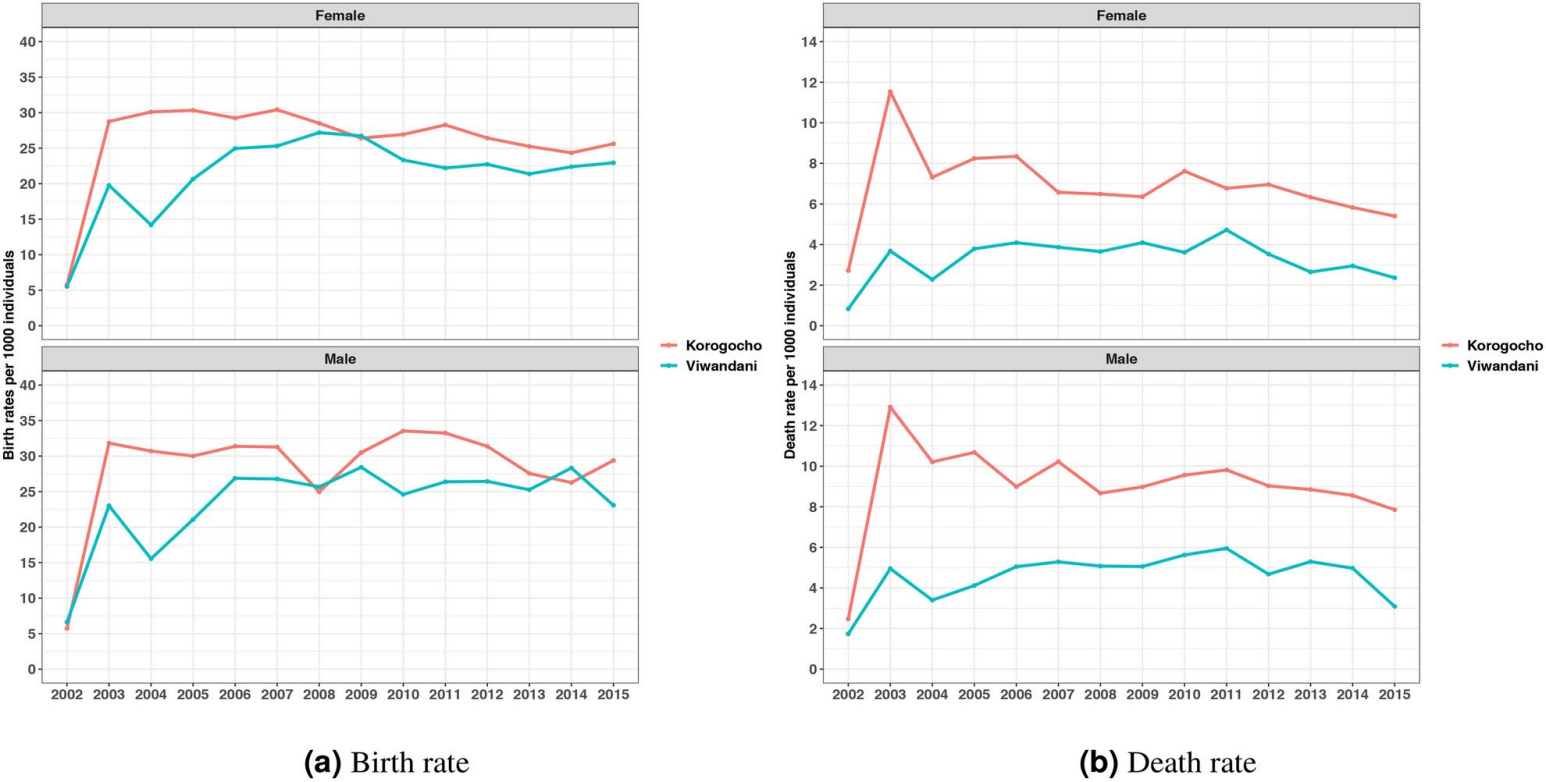
Annual birth and death rates per 1000 individuals within the Nairobi Urban Demographic Health and Surveillance Sites.

Figure 5a presents the annual birth and death rates per 1000 individuals within the two Demographic Surveillance Areas (DSAs) across the period from 2002 to 2015, broken down by gender. The trends indicate that both slums experienced similar patterns in birth rates over time, with notable fluctuations across the years. In both areas, birth rates for males generally appear higher than those for females. This pattern suggests gender-based differences in fertility behavior and dynamics within these urban populations. From the findings, there is consistent observation of higher birth rates in Viwandani compared to Korogocho. Similarly, in Fig. 5b, the death rates fluctuate over time, with both slums exhibiting similar trends, though the rates in Korogocho generally appear higher than in Viwandani. The death rates for males are consistently higher than those for females, suggesting gender differences in mortality within these populations. Notably, the trend indicates a gradual decline in death rates toward the later years. Supplementary Tables S3 and S4 provides further examination of birth, and death rates.

**Fig. 6:**
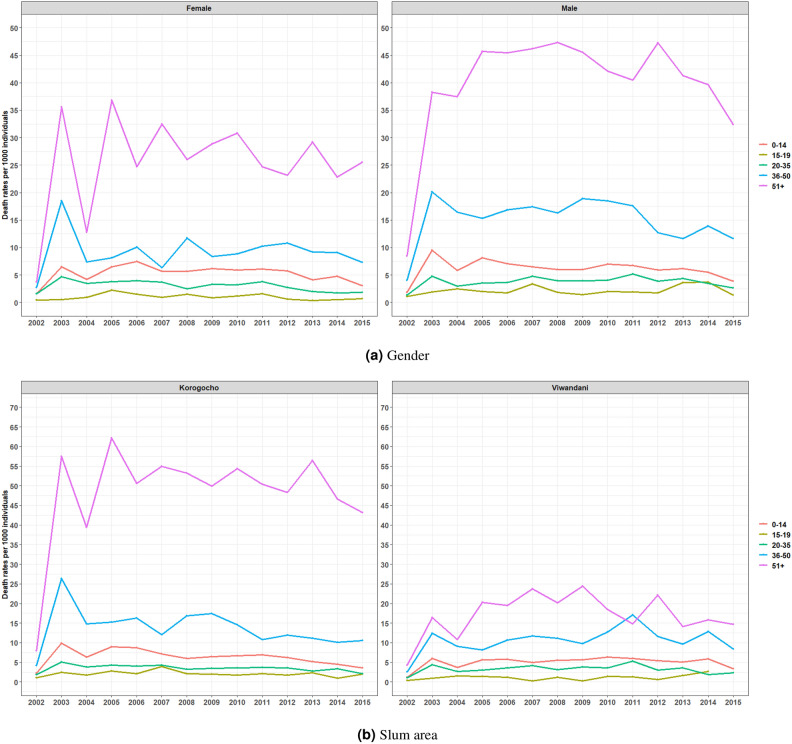
The age group specific death rates by gender and slum area.

Results in Fig. 6a illustrate death rates per age group per 1000 individuals in the two Nairobi’s slums from 2002 to 2015, disaggregated by gender and slum area. The findings show that the highest mortality rates are consistently observed in the 51+ age group for both males and females. For males, the highest death rates peaked around 2008 with gradual declines toward 2015. Among the females, a similar pattern is observed, but the rates are generally lower compared to males. The death rates in younger age groups (0–14, 15–19, and 20–35) are significantly lower and stable over time, with slight fluctuations. Additionally, Fig. 6b displays death rates in the two informal settlements: Korogocho (left panel) and Viwandani (right panel), where it is seen that death rates are consistently higher for older adults (51+) in both settlements, peaking in 2005 and generally declining over time, with Korogocho showing a steeper decline compared to Viwandani. In contrast, death rates for younger age groups are significantly lower and relatively stable throughout the period in the two areas. The age group 36–50 also experiences higher death rates compared to the younger groups (0–14, 15–19, and 20–35), particularly in the earlier years of the study, but sees a more pronounced decline in Korogocho as compared to Viwandani.

**Fig. 7:**
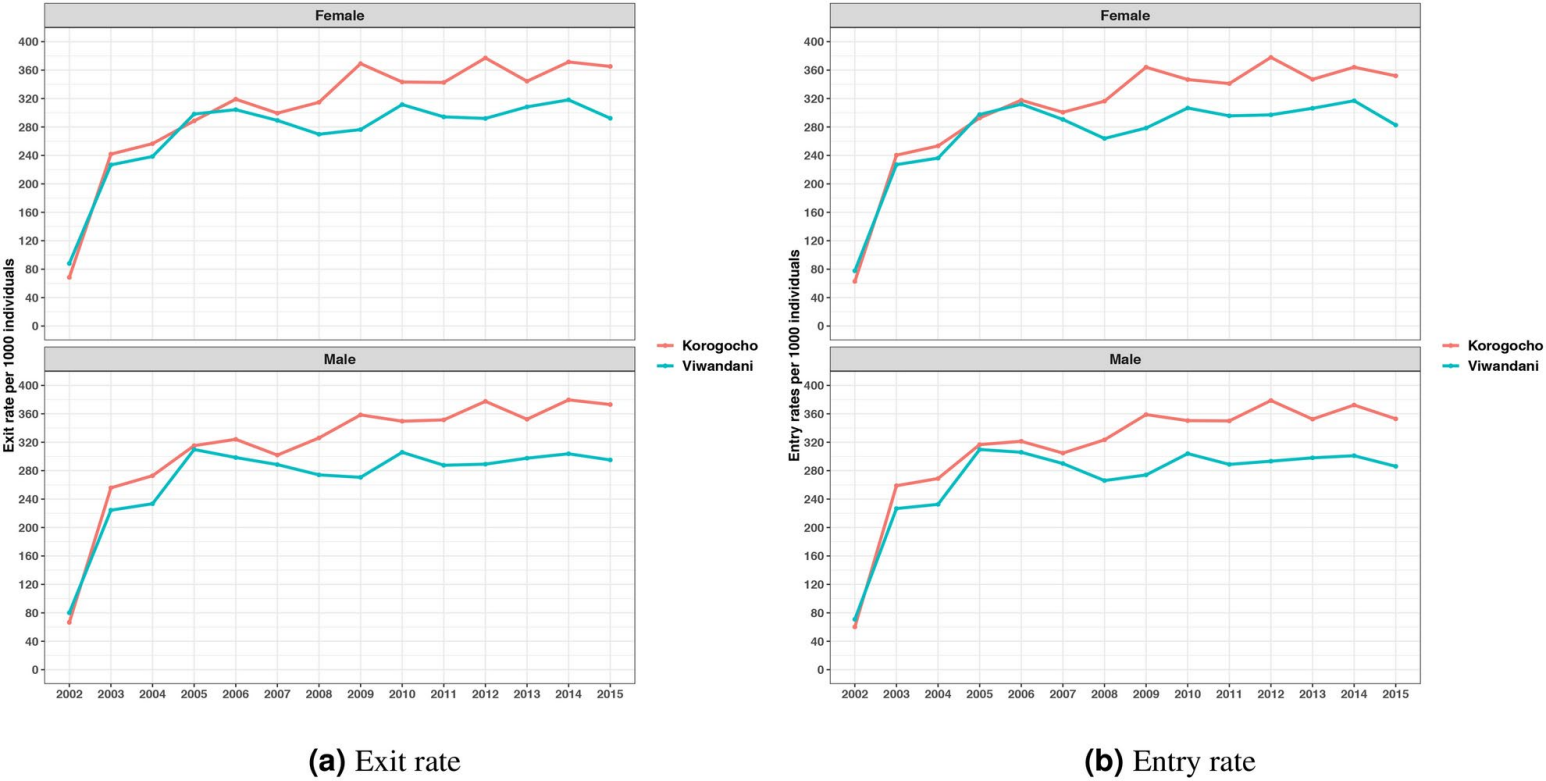
Annual entry and exit rates per 1000 individuals within the Nairobi Urban Demographic Health and Surveillance Sites.

In terms of exit rates which suggests the termination of residence in a particular household, Fig. 7a shows that there is a fluctuation in exit rates over time, with both slums exhibiting similar trends. A notable disparity emerged between the two slum areas, with Korogocho demonstrating consistently higher exit rates compared to Viwandani. Additionally, a gender-based analysis reveals that exit and entry rates are consistently high among males compared to their female counterparts. Figure 7b shows the indicate entry rates trends, which represent movement of individuals into new households. Generally, the entry rates fluctuate over time with notable peaks in 2012 and 2014. The rates for both males and females follow similar patterns, but Korogocho tends to have slightly higher entry rates compared to Viwandani. This pattern suggests that Korogocho may have experienced more movements into new households than Viwandani during this period. The sharp spikes observed in some years might indicate specific socio-economic factors or interventions that might have made more individuals to new households at different times.

The study also sought to identify the age group with the highest rates of exits and entries (movement to new households). Supplementary Figs. S1 and S2 illustrate exit and entry rates per 1000 individuals across different age groups from 2002 to 2015 in two informal settlements. In both settlements, the exit and entry rates generally increase over time, with noticeable differences between age groups. In Korogocho, older adults (51+) exhibit the highest exit and entry rates. The 15–19 age group has the lowest exit and entry rates, though these too increase over time. In Viwandani, both age groups 20–35 and 36–50 show the highest exit and entry rates, followed closely by the 15–19 age group. The findings indicate that exit and entry rates are higher in Korogocho for the older age group (51+), while in Viwandani, younger adults (20–35) and middle age people aged 35–50 exhibit the highest exit and entry. Additionally, Supplementary Figs. S3 and S4 provide findings about exit and entry disaggregated by gender. For females, the exit and entry rates show a general upward trend across all age groups, with the highest rates observed in the 15-19 age group, followed closely by the 20–35 age group. The older age groups, particularly 36–50 and 51+, exhibit lower exit and entry rates, though they still experience a gradual increase over time. For males, the exit and entry rates are notably higher across all age groups compared to females. The 35–50 age group shows the highest exit and entry rates. The 15–19 age group follows a similar trend but with the lowest rates. The results are indicative of the fact that males have higher exit and entry rates compared to females across all age groups. Supplementary Tables S3 and S4 provides further examination of exit and entry rates.

### Multi-state model analysis

**Table 2:** The observed transitions between residency events classification (N(%)).

Female							
From	To						
	Enumeration	Birth	Exit	Entry	Outmigration	Inmigration	Death
Enumeration	–	–	10,055 (46.3)	–	11,053 (51.0)	–	592 (2.7)
Birth	–	–	4,536 (45.6)	–	4,780 (48.0)	–	635 (6.4)
Exit	–	–	–	78,842 (100.0)	–	–	–
Entry	–	–	37,156 (57.4)	–	26,793 (41.4)	–	757 (1.2)
Outmigration	–	–	–	–	–	16,056 (100.0)	–
Inmigration	–	–	27,531 (39.7)	–	41,258 (59.5)	–	538 (0.8)
Death	–	–	–	–	–	–	–

Male							
Enumeration	–	–	13,882 (46.5)	–	15,791 (51.8)	–	832 (2.7)
Birth	–	–	4,904 (45.5)	–	5,171 (48.0)	–	696 (6.5)
Exit	–	–	–	93,539 (100.0)	–	–	–
Entry	–	–	43,057 (56.0)	–	32,752 (42.6)	–	1,093 (1.4)
Outmigration	–	–	–	–	–	18,934 (100.0)	–
Inmigration	–	–	32,282 (40.1)	–	47,375 (58.9)	–	775 (1.0)
Death	–	–	–	–	–	–	–

The movement patterns of individuals within the study population, as shown in Table 2, reveal comparable trends between females and males. Females recorded 10,055 instances of leaving the enumeration state to exit state, with 592 individuals progressing from enumeration to death. Similarly, males had 13,882 instances of moving from enumeration to exit, with 832 progressing from enumeration to death. Additionally, 757 females and 1093 males moved from entry to death, while 775 males and 538 females moved from in-migration to death. Given that most movements, except for death, are reversible, multiple occurrences of the same type of movement happened within individuals. The estimated mean duration spent in each state (sojourn time) is provided in Table 3. The results indicate that females spend an average of 813.3 days in the study population before experiencing another demographic event, whereas males take approximately 820 days. Specifically, females in the birth event remain in this state for an average of 680.3 days, compared to 640.2 days for males. This indicates that infants born in these Demographic Surveillance Areas generally do not experience a change in residence within their first two years of life, regardless of gender. Both females and males spend an average of 8 days in the exit state before moving to a new household. In contrast, individuals in the entry state remain there for an average of 535 days for females and 564 days for males before encountering another demographic event.

**Table 3:** The estimate of mean sojourn time.

States	Female			Male		
	Estimate	SE	95% CI	Estimate	SE	95% CI
Enumeration	813.3	5.8	(802.0, 824.8)	820.8	5.0	(811.1, 830.7)
Birth	680.3	198.8	(383.6, 1206.0)	640.2	254.2	(294.0, 1394)
Exit	8.2	0.1	(8.1, 8.2)	8.0	0.1	(7.9, 8.1)
Entry	535.8	2.1	(531.6, 540.0)	564.9	2.1	(560.8, 568.9)
Out-migration	588.7	4.7	(579.6, 598.0)	618.1	4.5	(609.3, 627.1)
Inmigration	566.7	2.5	(561.8, 571.6)	604.3	2.5	(599.4, 609.2)

**Fig. 8:**
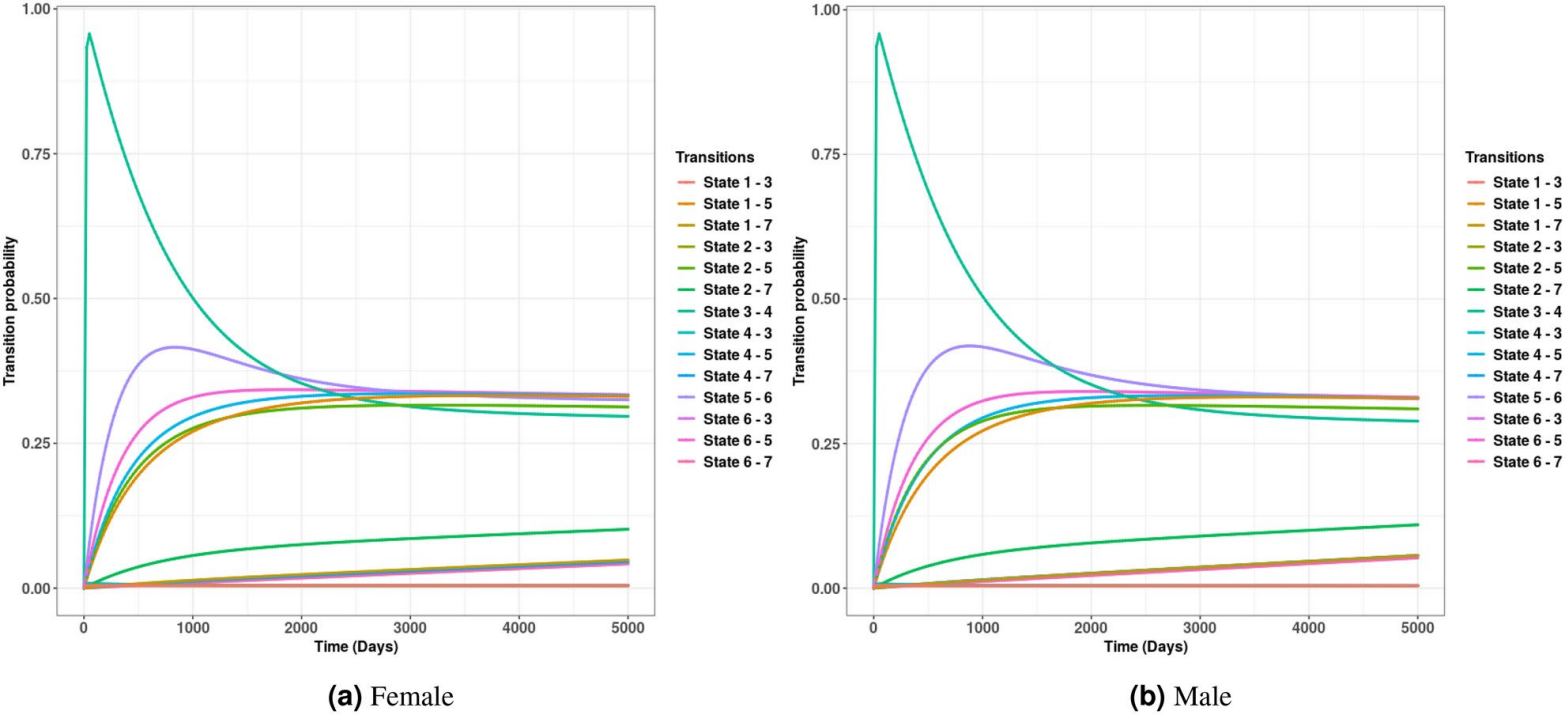
The transition probabilities. State 1: Enumeration, State 2: Birth, State 3: Exit, State 4: Entry, State 5: Out-migration, State 6: In-migration, State 7: Death.

In Fig. 8, we display the probabilities of moving between different states. We observed relatively higher probabilities of movement from entry to out-migration and from out-migration back to in-migration, with a peak occurring around year 2 (approximately 700 days) from the start of the NUHDSS for both females and males. The probabilities for these movements remain relatively constant across different states until year 13 (in 2015).

### Goodness of fit of the model

In order to establish how our fitted model accounts for the data, a goodness of fit is carried out. We carried out a Likelihood Ratio Test (LR Test) by comparing the null model (simple mode without covariates as the baseline model) and the full model with covariates. We also compared the Akaike Information Criterion (AIC) of the null model and the full model. The results are displayed in Table 4 for females and males. From the findings, the AIC for the male full model (AIC: 4,099,613) is lower than the AIC for the male null model (AIC: 4,107,351). This suggests that the more complex model with the covariates provides a better fit to the data than the simpler model. For the male gender, the likelihood ratio test outputs a chi-square statistic (Chisq = 7990.2) with 126 degrees of freedom, resulting in a $\text {p-value} <0.001$, which is highly statistically significant. This indicates that the full model fits the data significantly better than the simpler model. The large chi-square value and extremely low p-value suggest that the additional parameters in the full model provide substantial explanatory power beyond what is captured by the simpler model, making the more complex model a better choice for this analysis. Similar interpretations also apply to the female.

**Table 4:** Testing goodness of fit of the model.

Model	Number of parameters	Female model					Male			
		AIC	Likelihood-Ratio Test				AIC	Likelihood-Ratio Test		
			LogLik	df	Chisq	P-value		LogLik	Chisq	P-value
Simple model	14	3,414,222	− 1,707,097	126	5,915.8	<0.000	4,107,351	-2,053,662	7,990	<0.000
Model with covariates	140	3,408,558	− 1,704,139				4,099,613	-2,049,667

**Table 5:** The proportional hazard ratios (HR) and their corresponding 95% confidence intervals (CI) reported.

Variable	Comparison	Transition	Gender			
			Female		Male	
			HR	95% CI	**HR**	95% CI
Slum area	Viwandani vs Korogocho	Enumeration - Exit	1.390	(1.332, 1.450)	1.356	(1.306, 1.407)
		Enumeration - Outmigration	1.521	(1.461, 1.584)	1.656	(1.598, 1.716)
		Enumeration - Death	0.766	(0.627, 0.935)	0.661	(0.562, 0.779)
		Birth - Exit	0.918	(0.861, 0.980)	0.858	(0.807, 0.913)
		Birth - Outmigration	1.123	(1.053, 1.198)	1.187	(1.116, 1.262)
		Birth - Death	0.792	(0.664, 0.943)	0.803	(0.680, 0.948)
		Exit - Entry	0.896	(0.883, 0.910)	0.851	(0.838, 0.863)
		Entry - Exit	0.901	(0.881, 0.921)	0.904	(0.885, 0.923)
		Entry - Outmigration	1.173	(1.142, 1.205)	1.163	(1.135, 1.192)
		Entry - Death	0.858	(0.733, 1.003)	0.705	(0.619, 0.805)
		Outmigration - Inmigration	1.051	(1.015, 1.088)	1.030	(0.996, 1.064)
		Inmigration - Exit	1.008	(0.981, 1.036)	0.930	(0.907, 0.954)
		Inmigration - Outmigration	1.022	(0.999, 1.045)	1.033	(1.011, 1.055)
		Inmigration - Death	0.803	(0.660, 0.976)	0.600	(0.512, 0.704)
Ethnicity	Kikuyu vs Kamba	Enumeration - Exit	0.835	(0.786, 0.887)	0.856	(0.814, 0.900)
		Enumeration - Outmigration	0.809	(0.765, 0.855)	0.861	(0.823, 0.900)
		Enumeration - Death	0.980	(0.736, 1.304)	1.447	(1.144, 1.831)
		Birth - Exit	0.971	(0.889, 1.059)	1.019	(0.936, 1.109)
		Birth - Outmigration	0.775	(0.713, 0.843)	0.704	(0.649, 0.765)
		Birth - Death	1.034	(0.813, 1.317)	0.949	(0.750, 1.202)
		Exit - Entry	1.010	(0.989, 1.031)	1.173	(1.151, 1.195)
		Entry - Exit	1.023	(0.993, 1.054)	1.014	(0.986, 1.043)
		Entry - Outmigration	0.884	(0.854, 0.916)	0.965	(0.934, 0.996)
		Entry - Death	1.037	(0.834, 1.290)	1.421	(1.180, 1.711)
		Outmigration - Inmigration	0.957	(0.914, 1.003)	0.944	(0.906, 0.984)
		Inmigration - Exit	0.982	(0.949, 1.017)	0.906	(0.878, 0.936)
		Inmigration - Outmigration	0.867	(0.842, 0.891)	0.906	(0.883, 0.929)
		Inmigration - Death	1.014	(0.790, 1.302)	1.306	(1.063, 1.605)
	Luhyia vs Kamba	Enumeration - Exit	1.122	(1.044, 1.207)	1.134	(1.069, 1.203)
		Enumeration - Outmigration	1.020	(0.954, 1.092)	0.970	(0.918, 1.026)
		Enumeration - Death	1.139	(0.793, 1.635)	1.039	(0.770, 1.402)
		Birth - Exit	1.066	(0.965, 1.177)	1.065	(0.968, 1.171)
		Birth - Outmigration	1.015	(0.925, 1.112)	1.020	(0.936, 1.112)
		Birth - Death	1.168	(0.891, 1.532)	1.045	(0.804, 1.358)
		Exit - Entry	0.955	(0.933, 0.978)	0.988	(0.967, 1.009)
		Entry - Exit	1.014	(0.979, 1.050)	1.083	(1.050, 1.118)
		Entry - Outmigration	1.027	(0.988, 1.068)	1.090	(1.053, 1.129)
		Entry - Death	0.937	(0.720, 1.220)	1.224	(0.990, 1.514)
		Outmigration - Inmigration	1.047	(0.995, 1.102)	1.014	(0.968, 1.062)
		Inmigration - Exit	1.066	(1.027, 1.108)	1.137	(1.098, 1.177)
		Inmigration - Outmigration	1.047	(1.016, 1.080)	1.046	(1.016, 1.077)
		Inmigration - Death	0.915	(0.679, 1.233)	1.081	(0.843, 1.387)

Variables						
	Luo vs Kamba	Enumeration - Exit	0.947	(0.876, 1.023)	1.057	(0.990, 1.129)
		Enumeration - Outmigration	1.097	(1.023, 1.176)	1.090	(1.026, 1.159)
		Enumeration - Death	1.741	(1.248, 2.429)	2.146	(1.631, 2.823)
		Birth - Exit	0.811	(0.730, 0.901)	0.808	(0.729, 0.896)
		Birth - Outmigration	0.999	(0.909, 1.098)	1.099	(1.005, 1.201)
		Birth - Death	1.333	(1.024, 1.735)	1.587	(1.240, 2.032)
		Exit - Entry	1.008	(0.983, 1.035)	0.978	(0.955, 1.001)
		Entry - Exit	0.875	(0.842, 0.910)	0.927	(0.895, 0.961)
		Entry - Outmigration	1.168	(1.120, 1.218)	1.185	(1.140, 1.232)
		Entry - Death	1.604	(1.244, 2.068)	1.522	(1.223, 1.895)
		Outmigration - Inmigration	1.102	(1.044, 1.164)	1.015	(0.964, 1.068)
		Inmigration - Exit	0.919	(0.880, 0.959)	1.001	(0.962, 1.042)
		Inmigration - Outmigration	1.037	(1.003, 1.072)	1.051	(1.018, 1.086)
		Inmigration - Death	1.875	(1.422, 2.472)	1.778	(1.398, 2.260)
	Other vs Kamba	Enumeration - Exit	1.049	(0.977, 1.127)	1.116	(1.054, 1.181)
		Enumeration - Outmigration	0.701	(0.651, 0.754)	0.810	(0.766, 0.857)
		Enumeration - Death	0.728	(0.499, 1.062)	0.909	(0.669, 1.234)
		Birth - Exit	1.232	(1.120, 1.354)	1.167	(1.063, 1.281)
		Birth - Outmigration	0.723	(0.654, 0.799)	0.745	(0.678, 0.819)
		Birth - Death	0.742	(0.546, 1.008)	0.795	(0.598, 1.056)
		Exit - Entry	1.014	(0.991, 1.037)	1.074	(1.052, 1.096)
		Entry - Exit	1.120	(1.083, 1.158)	1.175	(1.141, 1.211)
		Entry - Outmigration	0.852	(0.819, 0.888)	0.978	(0.944, 1.012)
		Entry - Death	0.856	(0.657, 1.116)	0.861	(0.686, 1.081)
		Outmigration - Inmigration	1.083	(1.027, 1.142)	1.072	(1.024, 1.122)
		Inmigration - Exit	1.116	(1.074, 1.160)	1.164	(1.126, 1.204)
		Inmigration - Outmigration	0.945	(0.915, 0.976)	1.003	(0.975, 1.032)
		Inmigration - Death	0.719	(0.519, 0.995)	0.823	(0.628, 1.079)

Variables						
Area of birth	Rural Kenya vs Nairobi non-slum	Enumeration - Exit	1.024	(0.959, 1.092)	1.024	(0.966, 1.085)
		Enumeration - Outmigration	1.231	(1.155, 1.311)	1.175	(1.110, 1.244)
		Enumeration - Death	0.705	(0.557, 0.892)	0.725	(0.594, 0.885)
		Birth - Exit	0.743	(0.584, 0.944)	0.769	(0.605, 0.977)
		Birth - Outmigration	1.283	(1.069, 1.539)	1.105	(0.919, 1.330)
		Birth - Death	0.897	(0.507,1.587)	0.924	(0.532, 1.605)
		Exit - Entry	0.945	(0.921, 0.969)	0.994	(0.970, 1.018)
		Entry - Exit	0.981	(0.946, 1.018)	0.973	(0.939, 1.008)
		Entry - Outmigration	1.301	(1.243, 1.363)	1.257	(1.204, 1.312)
		Entry - Death	0.797	(0.624, 1.019)	0.696	(0.569, 0.852)
		Outmigration - Inmigration	0.844	(0.791, 0.901)	0.852	(0.802, 0.905)
		Inmigration - Exit	0.943	(0.895, 0.994)	0.917	(0.871, 0.964)
		Inmigration - Outmigration	1.028	(0.984, 1.075)	1.042	(0.997, 1.090)
		Inmigration - Death	0.769	(0.519, 1.140)	0.591	(0.442, 0.790)
	Same DSA slum vs Nairobi non-slum	Enumeration - Exit	0.969	(0.905, 1.038)	1.014	(0.954, 1.077)
		Enumeration - Outmigration	1.112	(1.040, 1.189)	1.099	(1.035, 1.167)
		Enumeration - Death	0.910	(0.714, 1.159)	0.876	(0.715, 1.072)
		Birth - Exit	0.744	(0.690, 0.802)	0.766	(0.713, 0.822)
		Birth - Outmigration	0.764	(0.701, 0.823)	0.747	(0.697, 0.800)
		Birth - Death	0.604	(0.498, 0.733)	0.631	(0.526, 0.756)
		Exit - Entry	0.978	(0.951, 1.005)	0.970	(0.945, 0.996)
		Entry - Exit	0.981	(0.943, 1.021)	0.937	(0.902, 0.974)
		Entry - Outmigration	0.890	(0.845, 0.936)	0.874	(0.833, 0.917)
		Entry - Death	1.197	(0.917, 1.564)	1.134	(0.915, 1.406)
		Outmigration - Inmigration	0.929	(0.867, 0.995)	0.993	(0.930,1.060)
		Inmigration - Exit	1.015	(0.950, 1.083)	0.949	(0.890, 1.012)
		Inmigration - Outmigration	0.797	(0.752, 0.845)	0.798	(0.753, 0.847)
		Inmigration - Death	0.959	(0.578, 1.593)	1.075	(0.744, 1.554)

Variables						
	Other vs Nairobi non-slum	Enumeration - Exit	1.020	(0.939, 1.108)	0.999	(0.927, 1.076)
		Enumeration - Outmigration	1.076	(0.991, 1.169)	1.171	(1.092, 1.256)
		Enumeration - Death	0.835	(0.611, 1.142)	0.819	(0.623, 1.078)
		Birth - Exit	0.968	(0.756, 1.239)	0.994	(0.782, 1.263)
		Birth - Outmigration	1.178	(0.942, 1.473)	1.219	(0.998, 1.490)
		Birth - Death	1.138	(0.635, 2.039)	0.950	(0.509, 1.776)
		Exit - Entry	0.919	(0.889, 0.952)	0.938	(0.907, 0.969)
		Entry - Exit	0.935	(0.890, 0.982)	0.935	(0.891, 0.980)
		Entry - Outmigration	1.006	(0.945, 1.071)	1.027	(0.969, 1.089)
		Entry - Death	0.838	(0.602, 1.166)	0.840	(0.639, 1.105)
		Outmigration - Inmigration	0.945	(0.863, 1.035)	1.042	(0.958, 1.133)
		Inmigration - Exit	1.026	(0.958, 1.098)	0.955	(0.893, 1.021)
		Inmigration - Outmigration	0.982	(0.927, 1.041)	1.060	(1.001, 1.122)
		Inmigration - Death	1.022	(0.625, 1.670)	0.709	(0.476, 1.056)
Age		Enumeration - Exit	0.993	(0.992, 0.994)	0.991	(0.990, 0.992)
		Enumeration - Outmigration	0.994	(0.993, 0.996)	0.994	(0.993, 0.995)
		Enumeration - Death	1.044	(1.039, 1.048)	1.039	(1.035, 1.043)
		Birth - Exit	0.994	(0.955, 1.034)	0.994	(0.948, 1.044)
		Birth - Outmigration	0.979	(0.932, 1.028)	0.988	(0.935, 1.044)
		Birth - Death	0.988	(0.870, 1.123)	0.992	(0.852, 1.155)
		Exit - Entry	1.001	(1.000, 1.002)	0.999	(0.999, 1.000)
		Entry - Exit	0.997	(0.996, 0.998)	0.993	(0.992, 0.994)
		Entry - Outmigration	0.991	(0.991, 0.992)	0.994	(0.993, 0.995)
		Entry - Death	1.051	(1.047, 1.055)	1.039	(1.035, 1.043)
		Outmigration - Inmigration	1.003	(1.002, 1.004)	1.001	(0.999, 1.002)
		Inmigration - Exit	0.997	(0.996, 0.998)	0.994	(0.993, 0.995)
		Inmigration - Outmigration	0.998	(0.997, 0.999)	0.996	(0.995, 0.997)
		Inmigration - Death	1.055	(1.050, 1.060)	1.043	(1.038, 1.048)

#### Female transitions

From the results in Table 5, we find that the slum area has a hazard ratio (HR) of 1.390 (95% CI 1.332–1.450) for females moving from enumeration (state 1) to exit (state 3). This means that females in Viwandani are 39.0% more likely to leave the enumeration state to exit state compared to females in Korogocho. This suggests that residing in Viwandani is associated with an increased likelihood of exiting a given household after being enumerated. Similar patterns are observed for individuals in Viwandani moving from enumeration to out-migration and from entry to out-migration. However, the results indicate that females in Viwandani have a 20.9% lower likelihood (HR: 0.792, CI 0.664–0.943) of moving from birth to death compared to those in Korogocho. A similar trend is seen among Viwandani residents for moving from enumeration to death and from in-migration to death.

In terms of ethnic group influence, the hazard ratio (HR) of 0.980 suggests that females from the Kikuyu ethnic group have approximately a 2.0% lower likelihood of moving from enumeration to death compared to the Kamba ethnic group over a given period. Additionally, since the 95% confidence interval (CI) includes the null value of 1, the results suggest that the association between the Kikuyu ethnic group and the likelihood of moving to death is not statistically significant at the 95% confidence level. A similar trend is observed when comparing other ethnic groups, such as Luhyia and Luo, against the Kamba ethnic group for both males and females.

When considering the place of birth, individuals born in rural settings, as opposed to those born in Nairobi’s non-slum areas, exhibit a higher likelihood of out-migrating from the enumeration state. Specifically, females born in rural settings are approximately 1.2 times more likely to out-migrate compared to females born in Nairobi’s non-slum areas. These trends are consistent among individuals born within the same DSA slum, indicating a comparable tendency for out-migration among those with rural origins. Age is observed to play a significant role in influencing movements between demographic states among females. The hazard ratio (HR) of 1.044 indicates that with each one-year increase in age, the likelihood of moving from enumeration to death rises by approximately 4.4%. The 95% confidence interval (CI) of [1.040, 1.048] shows the statistical significance of the association between age and the likelihood of dying. Conversely, movement from enumeration to out-migration exhibits a contrasting trend, with an HR of 0.994 indicating a 0.6% decrease in the likelihood of out-migrating with each additional year of age. This inverse relationship is also statistically significant, as evidenced by the HR falling within the 95% CI of [0.993, 0.996]. Similar patterns are observed across various other movements, including from birth to out-migration, birth to death, out-migration to in-migration, and vice versa. These findings emphasize the influence of age on the dynamics of demographic events, highlighting the importance of considering age as a critical factor in studies of population mobility and urbanization.

#### Male transitions

The results in Table 5 indicate that males in Viwandani are 35.6% more likely to move from enumeration to exit compared to males from Korogocho. This suggests that the slum area is associated with an increased likelihood of exiting a household after being enumerated. Similar patterns are observed for individuals in Viwandani, who are more likely to progress from enumeration to out-migration and from entry to out-migration. However, the results show that males from Viwandani have a 19.7% lower likelihood (HR 0.803, CI 0.680–0.948) of progressing from birth to death, respectively, compared to those in Korogocho. Comparable findings are observed for individuals in Viwandani in the movements from enumeration to death and in-migration to death. Additionally, males born in rural settings have a 1.2 times higher likelihood of out-migrating compared to their counterparts born in Nairobi non-slum areas. These trends are consistent among individuals born within the same DSA slum, indicating a similar propensity for out-migration among those with rural origins.

However, the hazard ratio (HR) of 1.5 suggests that males from the Kikuyu ethnic group, compared to the Kamba ethnic group, have approximately 1.45 times higher hazard of moving from enumeration to death over a given period. The 95% confidence interval of [1.144, 1.831] suggests that ethnic group remains a significant predictor of mortality probabilities among males. A similar trend is observed for the progression from birth to death and in-migration to death. This sequence is also seen among other ethnic groups, such as Luhya, Luo, and others, when compared to the Kamba ethnic group, across both genders. Age also plays a crucial role in influencing the likelihood of different demographic outcomes among males. The HR of 1.039 suggests a 3.9% increase in the likelihood of moving from enumeration to death with each additional year of age. Similar positive associations are also observed between entry and death as well as in-migration and death. However, there is a significant inverse relationship between age and the likelihood of moving from entry to out-migration. The HR of 0.994 implies a 0.6% decrease in the likelihood of out-migrating with each additional year of age.

From these findings it is important to note that both males and females in Viwandani are more likely to exit or out-migrate after enumeration compared to those in Korogocho, though they both have a lower likelihood of progressing from birth to death. Ethnic group and age are significant factors influencing these demographic movements: Kikuyu males show a higher hazard of death, while Kikuyu females have a slightly lower, though not statistically significant, likelihood of death compared to Kamba individuals. Additionally, older age increases the likelihood of mortality but decreases the likelihood of out-migration for both genders. These patterns emphasize the roles of slum area, ethnic background, and age in shaping demographic outcomes and highlight the importance of these factors in understanding population mobility and urbanization dynamics.

## Discussion

This study’s objective was to describe the changes in demographic events in the Demographic Surveillance Area (DSA), interpret these changes in relation to the population demographics, and inform policy in regards to the urbanization trends using residency data from the Nairobi Urban and Demographic Surveillance System (NUHDSS) from the year 2002 to the year 2015. In this study, a continuous time homogeneous multi-state Markov model is developed to effectively capture and model residency events, that include births, deaths, in-migrations, and out-migrations that directly impact the population dynamics including population growth, and population decline.

The study observed significant findings to evaluate out-migration and in-migration based on age, gender, ethnicity, place of birth, and slum area. It is established that there is relatively young median age (21–23 years) and the high proportion of individuals born in rural areas. This is a clear reflection of the early stages of transition, where rural-urban migration is a key driver of population growth in urban slums^24^. This migration pattern aligns with the urbanization processes described in both the Demographic Transition Theory and Urban Transition Theory, which highlights the shift from rural to urban living as populations seek better economic opportunities in cities, often resulting in rapid urban growth and the expansion of informal settlements^12–14^. However, it is also noteworthy that a significant proportion of the population reported to have been born within the same DSA slum and some originating from other slums in Nairobi. This is indicative of the fact that, a considerable segment of the population is potentially affected by urban poverty. This highlights the significant disadvantages the urban poor face with respect to access to vital services such health services. Studies by Fotso et al.^32^ and APHRC^33^ have relatively come up with similar findings thereby raising questions about urban poverty and its effects on health and social service delivery. These findings highlight the need to consider both the demographic transitions within the slums and the broader urban transition processes. The relatively stable median age and the predominance of younger individuals suggest that these slums are still experiencing significant population growth driven by both natural increase and continued in-migration, reflecting a transitional phase in both demographic and urban terms. Additionally, the ethnic composition of the population, with Kamba and Kikuyu being the predominant groups in Viwandani and Korogocho, respectively, also plays a significant role in shaping the socio-economic landscape of these slums. The concentration of certain ethnic groups in specific areas may be attributed to social networks and community ties that influence migration and settlement patterns^34^. This is consistent with the studies carried out by Pardede et al.^34^ and Zulu et al.^24^ on urbanization, which suggests that migrants often settle in areas where they have existing social connections, thereby reinforcing ethnic clustering within urban environments.

The observed trends in birth and death rates within the Nairobi slums of Korogocho and Viwandani from 2002 to 2015 offer important insights into the demographic and socio-economic dynamics of these populations. The fluctuations in birth rates, with consistently higher rates for males and pronounced peaks in 2012, suggest underlying gender-based differences in fertility behaviors. The higher birth rates in Viwandani compared to Korogocho could be linked to various socio-economic or environmental factors, such as differences in access to healthcare, employment opportunities, or cultural practices influencing reproductive decisions. These findings align with existing literature that emphasizes how urban slums, despite their proximity to urban centers, often exhibit distinct reproductive patterns influenced by the socio-economic conditions of the residents^35–37^. On the other hand, the trends in death rates, with higher mortality consistently observed in males and older adults, mirror broader patterns seen in urban slum environments, where men often face higher risks due to factors like occupational hazards, substance abuse, and violence^7,38^. The observed decline in death rates over time, particularly in the older age groups, could reflect improvements in healthcare access or targeted public health interventions within these communities, although the persistent higher death rates in Korogocho compared to Viwandani suggest that local conditions still play a significant role. These patterns are consistent with the literature, which notes that urban slums often experience varying degrees of health outcomes based on local environmental and socio-economic factors^39^. Furthermore, disaggregation of death rates by age group further reveals that older adults (51+) are particularly vulnerable, with the highest mortality rates observed in this group. The peak around 2008, followed by a gradual decline, may be indicative of specific health crises or broader socio-economic changes impacting the slum populations during that period. Meanwhile, the relatively stable and lower death rates among younger age groups (0–14, 15–19, and 20–35) could be a reflection of demographic resilience, possibly supported by community-based health initiatives aimed at reducing child and youth mortality^7,24^.

This study’s findings indicate that the migration patterns in Korogocho and Viwandani are characterized by consistently negative net migration rates and significant out-migration, align with broader socio-economic challenges commonly found in urban slums. The negative migration rates across most age groups and the sharper out-migration among older adults, particularly those aged 36–50 and 51+, suggest that these populations are likely driven away by adverse living conditions, economic instability, and perhaps the lack of opportunities in these areas. These findings resonate with existing literature, which shows that urban slum dwellers often face intense socio-economic pressures, such as unemployment, inadequate housing, and poor access to services, leading to high levels of mobility as individuals seek better living conditions elsewhere^8,24,34^. The higher net migration rates observed among younger individuals of the ages 15–19 and 20–35, suggest a contrasting trend where younger populations are moving into these slums, possibly due to the lower cost of living and proximity to economic opportunities in Nairobi’s industrial zones. This aligns with studies that have shown that young people, especially those migrating from rural areas, often move to urban slums in search of jobs or education, despite the challenging living conditions^6,8,25,32^. The differences in migration trends between Korogocho and Viwandani, with Viwandani showing more volatility, may reflect varying local factors, such as differences in economic opportunities, infrastructure, and social networks. In particular, Viwandani being adjacent to the industrial areas attracts more young people seeking employment opportunities as compared to Korogocho. The overall increase in net migration among younger age groups towards the end of the study period might indicate a growing attraction of these areas to the youth, possibly due to evolving economic conditions or social dynamics within the slums.

Internal movements (exit and entry) have also been analysed to draw insights into the dynamics of household mobility in the Nairobi slums of Korogocho and Viwandani from 2002 to 2015. Consistently higher exit rates in Korogocho compared to Viwandani reflect underlying socio-economic disparities between the two areas, with Korogocho facing more significant challenges such as higher crime rates, poorer living conditions, or greater economic instability, prompting residents to move out more frequently. This is consisted with studies in the literature that suggest urban slums, though similar in some respects, can exhibit varying levels of vulnerability and resilience based on local conditions^35,40^. In terms of gender differences in exit and entry rates, males tend to show consistently higher rates than females, pointing to the differing roles and responsibilities often assigned to men and women within these communities. The higher internal mobility among men could also be linked to cultural norms or economic pressures that compel them to seek employment opportunities and start their own households. In 2012 and 2014 there existed sharp spikes in exit and entry rates and may point to the government policies on the slum upgrading program about development of new housing units that necessitated movement into new households^41^. Regarding age-based differences, the high exit and entry rates among older adults (51+) in Korogocho could be due to factors such as the need for care or support that prompts movement into households of younger family members, or health-related issues necessitating relocation. In contrast, the higher internal mobility among younger adults (20–35) in Viwandani might reflect the pursuit of economic opportunities, as this age group is typically the most active in seeking employment or better living conditions^42^. The lower mobility rates among the 15–19 age group in both areas could indicate that younger individuals are more likely to remain within their original households, possibly due to dependence on family support or limited economic opportunities at this age.

The multi-state Markov model has been used to understand the progression of events by tracking how individuals move across different intermediate states in continuous time. This model provides deeper insights into complex event patterns, making it an effective tool for studying demographic changes. The multi-state model is increasingly used in demographic studies because it can capture the movement between various states that represent different stages in a person’s life or residency patterns^43^. The results from the analysis of the multi-state model provide insights into the factors influencing movements between demographic states. The findings highlight the significant influence of various demographic and socio-economic factors on these movements. For instance, the area of residence is crucial, with both females and males from Viwandani exhibiting a higher likelihood of out-migrating compared to those from Korogocho. This association remains significant, suggesting that the slum area independently influences residency changes, possibly reflecting differences in socio-economic conditions and access to opportunities between these two areas.

Moreover, ethnicity emerges as a significant predictor of residency patterns, with individuals from certain ethnic groups such as Kikuyus, Luos, and Luhyias showing higher likelihoods of dying compared to the Kamba ethnic group. This emphasizes the importance of addressing socio-cultural factors and disparities in health outcomes when designing interventions aimed at improving health and well-being within the population. Additionally, age consistently influences demographic movements, with older individuals (both males and females) showing a higher likelihood of dying while also exhibiting a lower likelihood of out-migrating. Another notable finding is that the impact of socio-demographic factors such as slum area, ethnic group, place of birth, and age on the rate of moving to the next demographic event is more pronounced when considering movements from enumeration to out-migration and from in-migration to out-migration for both males and females^44^. This pattern may again be partly attributed to economic factors. In addition, both males and females tend to out-migrate upon entry into a new household, further signaling the compounding effect of socio-demographic, economic, and health factors^44,45^. Length of stay (LoS) in a particular demographic state is a crucial measure for effective urban planning. Our estimates of the mean length of stay in enumeration and birth align with findings from Fotso et al.^46^. The authors observed that, on average, it takes approximately two years after birth before an individual out-migrates or exits a household to enter another household. However, a different pattern is observed for exit and entry, which are associated with shorter lengths of stay for both males and females, reflecting the typical movement between households. These insights are critical for planning resources, such as housing and social services, as they highlight the dynamic nature of household compositions and the frequent movements individuals undergo within urban environments.

The demographic events and dynamics highlighted in this study provide crucial insights for policymakers and researchers in tailoring targeted health interventions and policies to address the specific demographic and gender-related needs within the study areas. Given the findings of this study, it is clear that urban slums like Korogocho and Viwandani face significant challenges related to migration, demographic shifts, and socio-economic disparities. Policy makers should prioritize targeted interventions that address the root causes of these issues, particularly focusing on improving living conditions, enhancing access to essential services, and creating economic opportunities within these areas. Specifically, initiatives aimed at reducing crime rates, upgrading housing infrastructure, and providing better access to healthcare and education could help stabilize these communities. The study also highlights the importance of understanding the socio-cultural dynamics within these communities, especially in relation to ethnicity and age. Policy makers should consider designing culturally sensitive health and social programs that address the specific needs of different ethnic groups and age cohorts. For instance, targeted health interventions for older adults, who exhibit higher mortality rates, and economic support for younger adults, who are more mobile, could help address the specific challenges faced by these groups. Moreover, the significant role of ethnicity in influencing residency patterns suggests that policy makers should engage with community leaders from various ethnic groups to ensure that interventions are inclusive and effectively meet the needs of all residents.

Furthermore, these findings offer insights into the demographic dynamics and health outcomes within these communities, emphasizing the need for targeted interventions and policies to address gender-specific disparities and promote equitable healthcare access and reproductive health services. Our findings have important policy implications for health planning in Africa. First, high rates of migration in urban African cities have the potential to negatively impact access to health care. Urban migrants often face barriers in accessing healthcare services, leading to disparities in health outcomes and poor quality of life, especially among vulnerable populations such as children, women and the elderly^47^. In addition, the likelihood of increased spread and outbreaks of infectious diseases such as diarrhoea, cholera, and others is heightened in cities with high migration due to overcrowding, poor sanitation, and compromised access to safe water. Planners can use the estimates of net migration to ensure adequate health services are available in the city. Other anticipated health challenges of migration in cities include increased risk of poor nutrition, non-communicable diseases and mental health challenges due to the strenuous living conditions of migrants. Our findings provide benchmark estimates of key demographic outcomes that will contribute to planning for urban poor and cities in Africa. Finally, future research should build on the findings of this study by exploring the underlying factors that drive the observed demographic and migration patterns in greater detail. Longitudinal studies that track individuals over time could provide greater insights into how socio-economic, environmental, and policy changes impact population dynamics in urban slums. Additionally, further research is needed to understand the impact of specific interventions, such as slum upgrading programs and public health initiatives, on migration patterns and demographic changes within these communities.

The strengths of our study lies on its pioneering nature of evaluating multiple covariates simultaneously in a multi-state model, allowing for a comprehensive assessment of the effects of these covariates on demographic events transitions. This approach provides a better understanding of the factors driving population dynamics within the DSA. The use of hazard ratios (HR) with corresponding confidence intervals (CI) adds robustness to the analysis, allowing for the assessment of the statistical significance of the associations. Thus, the results enhance the reliability of the findings and provides a more comprehensive understanding of the relationships underlying the demographic events. However, this study is not without limitations, the observational nature of the study limits the ability to establish causal relationships between the covariates and demographic events transitions. While associations can be identified, causality cannot be inferred without further experimental or longitudinal evidence. Despite this constraints, the study’s thorough investigation of diverse factors influencing demographic events transitions forms a sturdy groundwork for targeted interventions to improve the quality of life in slum dwelling.

## Conclusion

This study challenges the common notion that slums are temporary and transient by revealing the persistence of these areas as long-term dwelling places. The findings suggest that rising poverty levels contribute to this permanence, as many residents remain in these settlements due to economic necessity rather than viewing them as temporary stops on the path to better living conditions. Additionally, the study highlights that a significant proportion of slum residents are migrants, which indicates that population growth in these areas is driven by individuals seeking employment opportunities in nearby industries. This influx emphasizes the critical role of economic factors in urban population dynamics, as people move to slum areas in search of jobs and the potential for improved living standards. From a modeling perspective, the multi-state model analysis provides insights into the complex demographic dynamics within the DSA. The results reiterate the significant influence of factors such as slum area, gender, ethnicity, place of birth, and age on demographic movements. These findings have important implications for public health interventions and policy efforts aimed at improving health outcomes, particularly in the urban slum settings of Viwandani and Korogocho.

The comprehensive analysis of demographic events in this study is essential for several reasons. First, it addresses a significant information gap by offering detailed insights into the prevalence and patterns of key demographic events, such as birth rates, death rates, and migration within the population. Second, this analysis is crucial for identifying marginalized segments of the population that may face disproportionate challenges related to migration and other demographic factors. The insights gained from this study can inform the development of targeted interventions, leading to more efficient resource allocation to meet the unique needs of these vulnerable groups. Ultimately, advocating for holistic strategies to address migration-related issues and population demographics is critical for advancing the overall welfare and stability of the population in these urban slum areas.

## Supplementary Information

Supplementary Information.

## Data Availability

R scripts presented used this study are available from GitHub repository and can be accessed through this link. The data can be accessed through microdata-portal, contingent upon proper approvals. However, the simulated data area available through this link.
