# Supplementary material for: Understanding demographic events and migration patterns in two urban slums of Nairobi City in Kenya
Source: Sci Rep. 2024 Nov 21;14:28852. doi: 10.1038/s41598-024-79895-x (PMC11582661; doi:10.1038/s41598-024-79895-x)
Supplement: Supplementary file 1 — Supplementary Information. [file 41598_2024_79895_MOESM1_ESM.docx]

**Understanding demographic events and migration patterns in two urban slums of Nairobi City in Kenya**

Evans Omondi^1,2, *^, Samuel Iddi^1,3^, Sharon Chepkemoi^1^, Bylhah Mugotitsa^1,4^, Steve Cygu^1^, Boscow Okumu^1,5^, Abdhalah Ziraba^1^, Damazo T. Kadengye^1^, and Agnes Kiragga^1^

*^1^African Population and Health Research Center (APHRC), Nairobi, Kenya.*

*^2^Institute of Mathematical Sciences, Strathmore University, Nairobi, Kenya.*

*^3^Department of Statistics and Actuarial Science, University of Ghana, Legon-Accra, Ghana.*

*^4^Strathmore University Business School, Strathmore University, Nairobi, Kenya.*

*^5^Environment for Development Initiative, Department of Economics, University of Nairobi, Nairobi, Kenya.*

[*^*^evansotieno@aims.ac.za*](mailto:*evansotieno@aims.ac.za)*.*

**Table S1:** **Descriptive statistics of the participants' characteristics from the year 2002 to 2015 (%).**

| **Year** | **2002** | **2003** | **2004** | **2005** | **2006** | **2007** | **2008** | **2009** | **2010** | **2011** | **2012** | **2013** | **2014** | **2015** | **P-value** |  |
| --- | --- | --- | --- | --- | --- | --- | --- | --- | --- | --- | --- | --- | --- | --- | --- | --- |
|  |  |  |  |  |  |  |  |  |  |  |  |  |  |  |  |  |
| **N** | **52,278** | **36,517** | **46,839** | **36,277** | **35,250** | **35,483** | **37,098** | **38,885** | **40,240** | **41,306** | **37,595** | **35,792** | **35,264** | **34,234** |  |  |
|  |  |  |  |  |  |  |  |  |  |  |  |  |  |  |  |  |
| **Slum area** | | | | | | | | | | | | | | |  |  |
| Korogocho | 47.1 | 37.5 | 35.3 | 41.8 | 43.8 | 42.4 | 43.2 | 44.1 | 39.5 | 39.0 | 39.7 | 38.8 | 38.4 | 35.7 | <0.001 |  |
| Viwandani | 52.9 | 62.5 | 64.7 | 58.2 | 56.2 | 57.6 | 56.8 | 55.9 | 60.5 | 61.0 | 60.3 | 61.2 | 61.6 | 64.3 |  |  |
| **Gender** | | | | | | | | | | | | | | | |  |
| Female | 42.5 | 43.8 | 44.0 | 45.6 | 45.4 | 45.3 | 45.5 | 45.7 | 45.4 | 45.6 | 45.5 | 45.9 | 45.7 | 46.8 | <0.001 |  |
| Male | 57.5 | 56.2 | 56.0 | 54.4 | 54.6 | 54.7 | 54.5 | 54.3 | 54.6 | 54.4 | 54.5 | 54.1 | 54.3 | 53.2 |  |  |
| **Ethnicity** | | | | | | | | | | | | | | | |  |
| Kamba | 24.0 | 27.7 | 28.7 | 26.6 | 25.4 | 24.5 | 24.6 | 25.3 | 26.4 | 25.7 | 26.1 | 26.0 | 26.3 | 27.5 | <0.001 |  |
| Kikuyu | 31.2 | 26.6 | 26.3 | 26.5 | 26.6 | 28.3 | 28.5 | 29.3 | 28.3 | 27.2 | 25.7 | 24.8 | 23.4 | 21.7 |  |  |
| Luhyia | 14.1 | 15.0 | 14.9 | 15.3 | 15.8 | 15.9 | 16.4 | 16.4 | 16.9 | 17.4 | 18.3 | 18.8 | 19.6 | 19.5 |  |  |
| Luo | 16.8 | 17.4 | 16.3 | 17.1 | 17.3 | 16.3 | 15.6 | 13.9 | 12.7 | 13.0 | 13.1 | 13.1 | 12.8 | 13.1 |  |  |
| Other | 14.0 | 13.4 | 13.8 | 14.6 | 15.0 | 15.0 | 14.9 | 15.1 | 15.8 | 16.7 | 16.9 | 17.3 | 17.8 | 18.2 |  |  |
| **Area of birth** | | | | | | | | | | | | | | | |  |
| Nairobi non-slum | 12.2 | 8.7 | 7.4 | 6.5 | 6.2 | 6.1 | 6.7 | 7.8 | 7.9 | 8.9 | 9.5 | 9.9 | 10.6 | 10.2 | <0.001 |  |
| Rural Kenya | 54.7 | 62.3 | 65.3 | 67.5 | 67.9 | 68.8 | 69.9 | 71.0 | 72.4 | 72.5 | 71.6 | 71.9 | 70.8 | 71.6 |  |  |
| Same DSA slum | 22.5 | 20.1 | 18.9 | 18.8 | 19.0 | 18.9 | 17.6 | 15.2 | 14.3 | 13.5 | 13.8 | 12.9 | 13.2 | 12.5 |  |  |
| Other places | 10.7 | 8.90 | 8.4 | 7.2 | 6.9 | 6.2 | 5.8 | 6.0 | 5.3 | 5.1 | 5.1 | 5.3 | 5.4 | 5.6 |  |  |
| **Age (M[IQR])** | 23 (12.5-33.5) | 22 (12.0-32-0) | 22 (11.5-32.5) | 21 (10.5-31.5) | 21 (10.5-31.5) | 21 (10.5-31.5) | 21 (10.5-31.5) | 21 (10.5-31.5) | 21 (10.0-32.0) | 21 (10.5-31.5) | 22 (11.5-32.5) | 22 (11.5-32.5) | 21 (10.5-31.5) | 21 (10.5-31.5) | <0.001 |  |

Table **S1** provides descriptive statistics of the cohort from the NUHDSS for the period 2002 to 2015. Pearson's chi-squared test for the categorical variable(s) and Kruskal-Wallis rank sum test for continuous variable(s) are performed. The results are stratified by gender, ethnicity, and type of area in which an individual was born, offering insights on the longitudinal perspective of the population demographics and potential migration patterns. The gender distribution among participants shifted slightly over the 14-year period, with females increasing from 42.5% to 46.8% of the study population. The significant p-value (< 0.001) suggests that these changes are unlikely to be due to random variation alone and instead may reflect real population dynamics over time. The findings show variations in ethnic representation, with Kikuyu ethnicity experiencing a marginal rise from 24.0% to 27.5%. The Luo ethnicity saw a decrease from the highest rate of 17.4% to the lowest rate of 12.8%. These shifts may be reflective of migration patterns, possibly driven by economic opportunities or disparities in birth and death rates among ethnic groups. The statistical significance (p-value < 0.001) shows the potential implications of these changes for targeted public health interventions and resource allocation.

There is a discernible pattern in the birthplace of participants, with a consistent majority being born in rural Kenya. However, there is a considerable proportion of the population reported to have been born within the same DSA slum, with 2002 recording the highest percentage of 22.5% and 2015 recording the lowest rate at 12.9%. The rural birthplace percentage remained relatively stable with 2011 recording the highest rate of individuals born in rural Kenya at 72.6% and the least being 54.6% in 2002. This disparity raises questions about rural-urban migration trends and their effects on health and social service delivery in urban settings. Over the span of 2002 to 2015, the median age of the population has shown a fluctuation between 21 years and 23 years. The IQR also shows a slight narrowing, indicating a more consistent age range among the population over time. The statistical significance (p < 0.001) confirms that these changes are meaningful and not due to random variation.

**Table S2: Annual in-migration, out-migration and net migration rates per 1000 individuals**

| **Year** | **Slum area** | | | | | | **Gender** | | | | | | **Overall** | | |
| --- | --- | --- | --- | --- | --- | --- | --- | --- | --- | --- | --- | --- | --- | --- | --- |
|  | **Korogocho** | | | **Viwandani** | | | **Male** | | | **Female** | | |  |  |  |
|  | **In** | **Out** | **Net** | **In** | **Out** | **Net** | **In** | **Out** | **Net** | **In** | **Out** | **Net** | **In** | **Out** | **Net** |
| **2002** | 39.7 | 33 | 6.7 | 72 | 35.9 | 36.1 | 53.4 | 31.9 | 21.5 | 61.3 | 38.1 | 23.2 | 56.8 | 34.5 | 22.2 |
| **2003** | 341 | 326 | 14.3 | 390 | 351 | 38.1 | 351 | 343 | 7.5 | 398 | 340 | 57.1 | 371 | 342 | 29.2 |
| **2004** | 364 | 380 | -16.1 | 345 | 515 | -170 | 342 | 469 | -127 | 364 | 465 | -101 | 352 | 467 | -115 |
| **2005** | 344 | 365 | -20.6 | 420 | 366 | 54.6 | 378 | 363 | 15.5 | 401 | 368 | 32.3 | 389 | 365 | 23.2 |
| **2006** | 327 | 370 | -43.4 | 389 | 353 | 36 | 358 | 358 | -0.1 | 366 | 363 | 2.6 | 362 | 360 | 1.2 |
| **2007** | 295 | 424 | -129 | 392 | 366 | 25.8 | 352 | 386 | -34 | 349 | 396 | -46.7 | 351 | 391 | -39.8 |
| **2008** | 341 | 350 | -8.9 | 414 | 328 | 86.5 | 384 | 339 | 45.4 | 381 | 335 | 45.1 | 383 | 337 | 45.3 |
| **2009** | 377 | 315 | 62.2 | 419 | 334 | 85.4 | 399 | 325 | 74.2 | 402 | 326 | 76.3 | 401 | 326 | 75.2 |
| **2010** | 340 | 354 | -14.3 | 364 | 359 | 4.4 | 357 | 353 | 4.1 | 351 | 363 | -11.6 | 354 | 357 | -3 |
| **2011** | 346 | 353 | -6.9 | 367 | 402 | -35.3 | 354 | 380 | -26 | 364 | 386 | -22.2 | 359 | 383 | -24.3 |
| **2012** | 261 | 399 | -138 | 339 | 406 | -67.2 | 309 | 401 | -92.1 | 308 | 407 | -99.4 | 308 | 404 | -95.4 |
| **2013** | 280 | 391 | -111 | 330 | 392 | -61.8 | 303 | 395 | -91.9 | 319 | 386 | -67.7 | 310 | 391 | -80.8 |
| **2014** | 288 | 371 | -83.4 | 324 | 395 | -71.5 | 303 | 389 | -86.7 | 318 | 382 | -63.5 | 310 | 386 | -76.1 |
| **2015** | 325 | 294 | 31.4 | 397 | 318 | 78.8 | 361 | 317 | 43.4 | 384 | 301 | 82.9 | 371 | 310 | 61.9 |

The results in supplementary Table **S2** present the net migration per 1000 individuals. The results show that there was a positive net migration in population per thousand in 2002 which dropped in 2004 for both Korogocho and Viwandani. The negative trend persisted in Korogocho for about 12 years. However, this was not the case in Viwandani as it depicted a positive trend up to 2010. Overall, from 2002 to 2015, the migration pattern shows significant fluctuation. In 2002 and 2003 there was a positive net migration of 22.2 and 29.2, respectively. However, in 2004, there was a substantial net loss of 115.4, indicating more people moved out than moved in. The subsequent years generally alternated between net gains and losses, with notable net gains in 2005 (23.2) and 2006 (1.2). In 2007, 2010, and 2011 there was a considerable net loss of 39.8, 3.0, and 24.3, respectively. Conversely, 2008 and 2009 showed positive net migrations of 45.3 and 75.2. The trend continued with a net decrease in 2012 (-95.4) and 2013 (-80.8), but 2015 ended on a positive note with net migrations of 61.9, reflecting more individuals moving into the region than leaving.

**Table S3: Annual birth and death, exit and entry rates per 1000 individuals within the Nairobi Urban Demographic Health and Surveillance Sites.**

| **Year** | **Slum area** | | | | **Gender** | | | | **Overall** | |  |
| --- | --- | --- | --- | --- | --- | --- | --- | --- | --- | --- | --- |
|  | **Korogocho** | | **Viwandani** | | **Male** | | **Female** | |  |  |  |
|  | **Birth** | **Death** | **Birth** | **Death** | **Birth** | **Death** | **Birth** | **Death** | **Birth** | **Death** |  |
| **2002** | 5.4 | 2.4 | 6.4 | 1.4 | 6.2 | 2.1 | 5.6 | 1.7 | 11.8 | 3.8 |  |
|  |  |  |  |  |  |  |  |  |  |  |  |
| **2003** | 22.7 | 9.2 | 26.7 | 5.4 | 26.3 | 7.9 | 23.1 | 6.6 | 49.5 | 14.6 |  |
|  |  |  |  |  |  |  |  |  |  |  |  |
| **2004** | 21.5 | 6.2 | 19.2 | 3.7 | 20.9 | 5.8 | 19.8 | 4.1 | 40.7 | 9.9 |  |
|  |  |  |  |  |  |  |  |  |  |  |  |
| **2005** | 25.2 | 7.9 | 24.3 | 4.6 | 24.8 | 6.9 | 24.7 | 5.7 | 49.5 | 12.5 |  |
|  |  |  |  |  |  |  |  |  |  |  |  |
| **2006** | 26.6 | 7.6 | 29.1 | 5.1 | 28.9 | 6.8 | 26.8 | 6 | 55.7 | 12.7 |  |
|  |  |  |  |  |  |  |  |  |  |  |  |
| **2007** | 26.2 | 7.1 | 30 | 5.3 | 28.7 | 7.4 | 27.5 | 5 | 56.2 | 12.4 |  |
|  |  |  |  |  |  |  |  |  |  |  |  |
| **2008** | 23.1 | 6.6 | 30 | 5 | 25.4 | 6.6 | 27.8 | 4.9 | 53.1 | 11.5 |  |
|  |  |  |  |  |  |  |  |  |  |  |  |
| **2009** | 25.1 | 6.8 | 30.8 | 5.1 | 29.3 | 6.8 | 26.6 | 5.1 | 55.9 | 11.9 |  |
|  |  |  |  |  |  |  |  |  |  |  |  |
| **2010** | 23.9 | 6.8 | 29 | 5.6 | 28.1 | 7.2 | 24.8 | 5.2 | 52.9 | 12.4 |  |
|  |  |  |  |  |  |  |  |  |  |  |  |
| **2011** | 24 | 6.5 | 29.7 | 6.5 | 29.1 | 7.5 | 24.6 | 5.5 | 53.6 | 13 |  |
|  |  |  |  |  |  |  |  |  |  |  |  |
| **2012** | 23 | 6.4 | 29.6 | 4.9 | 28.4 | 6.4 | 24.2 | 4.9 | 52.6 | 11.3 |  |
|  |  |  |  |  |  |  |  |  |  |  |  |
| **2013** | 20.5 | 5.9 | 28.5 | 4.9 | 26.2 | 6.7 | 22.9 | 4.1 | 49 | 10.8 |  |
|  |  |  |  |  |  |  |  |  |  |  |  |
| **2014** | 19.5 | 5.5 | 31.2 | 4.9 | 27.5 | 6.4 | 23.1 | 4.1 | 50.7 | 10.4 |  |
|  |  |  |  |  |  |  |  |  |  |  |  |
| **2015** | 19.6 | 4.7 | 29.6 | 3.5 | 25.3 | 4.8 | 23.9 | 3.4 | 49.2 | 8.2 |  |
|  |  |  |  |  |  |  |  |  |  |  |  |
| **Year** | **Exit** | **Entry** | **Exit** | **Entry** | **Exit** | **Entry** | **Exit** | **Entry** | **Exit** | **Entry** |  |
|  |  |  |  |  |  |  |  |  |  |  |  |
| **2002** | 67.5 | 61.4 | 83.2 | 73.5 | 74.2 | 66.2 | 77.9 | 70 | 75.8 | 67.8 |  |
|  |  |  |  |  |  |  |  |  |  |  |  |
| **2003** | 249 | 249.8 | 225.3 | 226.8 | 235.2 | 237.7 | 233.1 | 232.7 | 234.2 | 235.5 |  |
|  |  |  |  |  |  |  |  |  |  |  |  |
| **2004** | 264.9 | 261.3 | 235.5 | 234.1 | 246.2 | 244.4 | 245.6 | 243 | 245.9 | 243.7 |  |
|  |  |  |  |  |  |  |  |  |  |  |  |
| **2005** | 301.9 | 304.6 | 304.9 | 304.6 | 311.9 | 312.4 | 293.8 | 295.3 | 303.6 | 304.6 |  |
|  |  |  |  |  |  |  |  |  |  |  |  |
| **2006** | 321.6 | 319.5 | 301 | 308.5 | 309 | 312.2 | 311.4 | 314.8 | 310.1 | 313.4 |  |
|  |  |  |  |  |  |  |  |  |  |  |  |
| **2007** | 300.7 | 302.8 | 288.9 | 290.3 | 294 | 296 | 294 | 295.3 | 294 | 295.7 |  |
|  |  |  |  |  |  |  |  |  |  |  |  |
| **2008** | 320.5 | 319.9 | 272.2 | 265.1 | 295.3 | 289.4 | 290.6 | 288.1 | 293.1 | 288.8 |  |
|  |  |  |  |  |  |  |  |  |  |  |  |
| **2009** | 363.5 | 361.4 | 273.1 | 276 | 307.9 | 310 | 319.2 | 318.1 | 313.1 | 313.7 |  |
|  |  |  |  |  |  |  |  |  |  |  |  |
| **2010** | 346.6 | 348.7 | 308.3 | 305.2 | 322.6 | 321.7 | 324.6 | 323.2 | 323.5 | 322.4 |  |
|  |  |  |  |  |  |  |  |  |  |  |  |
| **2011** | 347.3 | 345.8 | 290.6 | 291.9 | 311.7 | 311.9 | 313.8 | 314 | 312.7 | 312.9 |  |
|  |  |  |  |  |  |  |  |  |  |  |  |
| **2012** | 377.3 | 378.3 | 290.4 | 295 | 322.8 | 325.7 | 327.8 | 331.1 | 325.1 | 328.2 |  |
|  |  |  |  |  |  |  |  |  |  |  |  |
| **2013** | 348.4 | 349.8 | 302.3 | 301.7 | 317.7 | 318.1 | 323.2 | 323.1 | 320.2 | 320.4 |  |
|  |  |  |  |  |  |  |  |  |  |  |  |
| **2014** | 375.7 | 368.4 | 310.1 | 308.1 | 331.7 | 327.3 | 339.7 | 336 | 335.3 | 331.2 |  |
|  |  |  |  |  |  |  |  |  |  |  |  |
| **2015** | 369.2 | 352.5 | 293.8 | 284.5 | 322.1 | 309.2 | 319.2 | 308.2 | 320.8 | 308.8 |  |
|  |  |  |  |  |  |  |  |  |  |  |  |

Supplementary Table **S3** presents the annual birth and death rates per 1,000 individuals within the two Demographic Surveillance Areas (DSAs) across the period from 2002 to 2015. It is observed that there is a fluctuation in the birth rates over the years. In 2002, the birth rate per 1000 individuals was 5.41 in Korogocho and 6.43 in Viwandani. Notably, Viwandani consistently exhibits higher birth rates compared to Korogocho throughout the years. However, both areas experienced an overall increase in birth rates from 2002 to 2006, reaching a peak in 2006 with 26.58 and 29.11 per 1000 individuals in Korogocho and Viwandani, respectively. Subsequently, a general declining trend is observed, with 2015 recording 19.63 and 29.59 in Korogocho and Viwandani, respectively. Similarly, death rates display temporal variations. The male gender generally maintains higher death rates compared to the female gender over the years. In 2002, death rates were 2.45 and 1.34 per 1000 individuals in male and female, respectively. Both genders experienced fluctuations in death rates, with males peaking at 7.94 in 2003, and females at 6.63 in the same year. The findings depicted in supplementary Table **S3** reveal a persistent trend wherein the rates of male births and deaths are consistently higher than their female counterparts. Overall, the birth rates fluctuated significantly, ranging from a low of 11.8 in the year 2002 to a high of 56.2 in 2007. Correspondingly, the death rates also varied, from a low of 3.8 in 2002 to a high of 14.6 in 2003. Generally, higher birth rates were observed in the mid-2000s, coinciding with moderate to high death rates, suggesting an overall net population growth despite the mortality rates. For instance, in 2007, a high birth rate of 56.2 was paired with a death rate of 12.4, indicating significant population growth. Conversely, the years with lower birth rates, such as 2002 with a birth rate of 11.8 and a death rate of 3.8, reflect smaller net population increases.

Moreover, the results in supplementary Table **S3** shows that across the years spanning from 2002 to 2015, both entry and exit rates exhibited an upward trend. Additionally, a notable disparity emerged between the two slum areas, with Korogocho demonstrating consistently higher entry and exit rates compared to Viwandani. Additionally, a gender-based analysis reveals that exit and entry rates are consistently high among males compared to their female counterparts. The supplementary Table **S4** provides further examination of population movements. Overall, the exit rates ranged from a low of 75.8 to a high of 335.3, while the entry rates varied from 67.8 to 331.2. Generally, entry rates closely mirrored exit rates, indicating a relatively balanced pattern of movement. For instance, in 2003, the exit rate was 234.2, nearly identical to the entry rate of 235.5. Similarly, in 2006, both exit and entry rates were in the 300s, specifically 303.6 and 304.6, respectively. There were instances where the exit rates slightly exceeded entry rates, such as in 2015 (320.8 exits vs. 308.8 entries), hinting at a potential net internal outflow.

**Table S4: Annual number of demographic events within the NUHDSS**

| **Calendar year** | **Demographic event** | **Total counts** | **Event incidences** | **Median [IQR]** |
| --- | --- | --- | --- | --- |
| **2002** | Birth | 619 | 143 | 4[3, 6] |
|  | Death | 199 | 98 | 2[1, 3] |
|  | Entry | 3,632 | 148 | 18[9, 31] |
|  | Exit | 4,088 | 151 | 17[10, 33] |
|  | In-migration | 2,968 | 152 | 13[8, 21] |
|  | Out-migration | 1,806 | 144 | 8[5, 15] |
| **2003** | Birth | 1,806 | 365 | 5[3, 6] |
|  | Death | 532 | 253 | 2[1, 3] |
|  | Entry | 9,329 | 365 | 18[11, 31] |
|  | Exit | 9,287 | 364 | 15[8, 29] |
|  | In-migration | 13,557 | 365 | 24[15, 46] |
|  | Out-migration | 12,502 | 365 | 19[10, 35] |
| **2004** | Birth | 1,907 | 359 | 5[4, 7] |
|  | Death | 462 | 246 | 1[1, 2] |
|  | Entry | 13,301 | 364 | 24[14, 44] |
|  | Exit | 13,451 | 365 | 19[11, 43] |
|  | In-migration | 16,519 | 366 | 27[17, 60] |
|  | Out-migration | 21,938 | 366 | 26[14, 64] |
| **2005** | Birth | 1,796 | 357 | 5[3, 6] |
|  | Death | 454 | 235 | 1[1, 2] |
|  | Entry | 12,626 | 365 | 21[11, 46] |
|  | Exit | 12,609 | 362 | 16[7, 46] |
|  | In-migration | 14,097 | 365 | 22[12, 46] |
|  | Out-migration | 13,266 | 358 | 18[10, 43] |
| **2006** | Birth | 1,963 | 365 | 5[4, 7] |
|  | Death | 449 | 221 | 1[1, 2] |
|  | Entry | 12,483 | 361 | 18[11, 40] |
|  | Exit | 12,328 | 360 | 15[8, 38] |
|  | In-migration | 12,752 | 363 | 17[10, 47] |
|  | Out-migration | 12,713 | 363 | 16[8, 37] |
| **2007** | Birth | 1,993 | 362 | 5[4, 7] |
|  | Death | 440 | 216 | 1[1, 2] |
|  | Entry | 11,886 | 364 | 18[10, 41] |
|  | Exit | 11,791 | 360 | 16[7, 39] |
|  | In-migration | 12,459 | 364 | 17[9, 44] |
|  | Out-migration | 13,876 | 364 | 19[10, 44] |
| **2008** | Birth | 1,971 | 364 | 5[4, 7] |
|  | Death | 427 | 224 | 1[1, 2] |
|  | Entry | 11,919 | 364 | 19[10, 40] |
|  | Exit | 12,149 | 364 | 16[9, 39] |
|  | In-migration | 14,201 | 365 | 23[13, 46] |
|  | Out-migration | 12,533 | 363 | 20[10, 45] |
| **2009** | Birth | 2,175 | 365 | 6[4, 7] |
|  | Death | 462 | 238 | 1[1, 2] |
|  | Entry | 14,158 | 365 | 22[13, 46] |
|  | Exit | 14,110 | 363 | 19[10, 47] |
|  | In-migration | 15,584 | 364 | 23[14, 56] |
|  | Out-migration | 12,673 | 365 | 16[9, 43] |
| **2010** | Birth | 2,128 | 363 | 6[4, 7] |
|  | Death | 498 | 243 | 2[1, 2] |
|  | Entry | 15,059 | 364 | 24[13, 51] |
|  | Exit | 15,109 | 359 | 20[10, 56] |
|  | In-migration | 14,264 | 364 | 20[11, 55] |
|  | Out-migration | 14,397 | 362 | 20[10, 58] |
| **2011** | Birth | 2,215 | 362 | 6[4, 8] |
|  | Death | 536 | 244 | 1[1, 2] |
|  | Entry | 14,954 | 363 | 21[12, 47] |
|  | Exit | 14,966 | 356 | 18[9, 54] |
|  | In-migration | 14,816 | 364 | 20[10, 49] |
|  | Out-migration | 15,831 | 365 | 20[9, 61] |
| **2012** | Birth | 1,978 | 364 | 5[4, 7] |
|  | Death | 425 | 239 | 1[1, 2] |
|  | Entry | 14,378 | 365 | 22[12, 53] |
|  | Exit | 14,200 | 359 | 17[9, 56] |
|  | In-migration | 11,601 | 360 | 15[8, 41] |
|  | Out-migration | 15,199 | 363 | 21[11, 59] |
| **2013** | Birth | 1,755 | 360 | 5[3, 6] |
|  | Death | 385 | 221 | 1[1, 2] |
|  | Entry | 12,883 | 364 | 22[11, 44] |
|  | Exit | 12,894 | 361 | 18[9, 43] |
|  | In-migration | 11,112 | 364 | 19[10, 38] |
|  | Out-migration | 14,012 | 364 | 22[12, 52] |
| **2014** | Birth | 1,787 | 363 | 4[3, 6] |
|  | Death | 367 | 225 | 1[1, 2] |
|  | Entry | 13,532 | 365 | 24[15, 48] |
|  | Exit | 13,720 | 362 | 23[12, 46] |
|  | In-migration | 10,932 | 359 | 18[10, 38] |
|  | Out-migration | 13,619 | 364 | 24[12, 50] |
| **2015** | Birth | 1,685 | 359 | 4[3, 6] |
|  | Death | 282 | 179 | 1[1, 2] |
|  | Entry | 12,241 | 363 | 17[8, 37] |
|  | Exit | 12,701 | 355 | 16[8, 38] |
|  | In-migration | 12,725 | 361 | 16[9, 33] |
|  | Out-migration | 10,608 | 349 | 12[5, 30] |

The results in Table **S4** show key residency events, including birth and death rates, migration patterns, and urbanization trends in the DSA. In 2002, the NUHDSS recorded 619 births, 199 deaths, 3,632 entries, 4,088 exits, 2,968 in-migrations, and 1806 out-migrations. Subsequent years reveal varying demographic trends, such as in 2009 with 2,175 births, 462 deaths, 14,158 entries, 14,110 exits, 15,584 in-migrations, and 12,673 out-migrations. These figures offer a detailed portrayal of the population dynamics over time. The birth rates within the NUHDSS exhibit a dynamic pattern across the years, ranging from 1,685 in 2015 to a peak of 2215 in 2011. Similarly, death rates display variability, reaching a low of 179 in 2015 and a high of 536 in 2011. Mobility trends within the DSA are apparent in the entry and exit numbers, fluctuating from a minimum of 619 entries in 2002 to a maximum of 9,329 entries in 2003, and from 9,287 exits in 2003 to 12,149 exits in 2008, respectively.


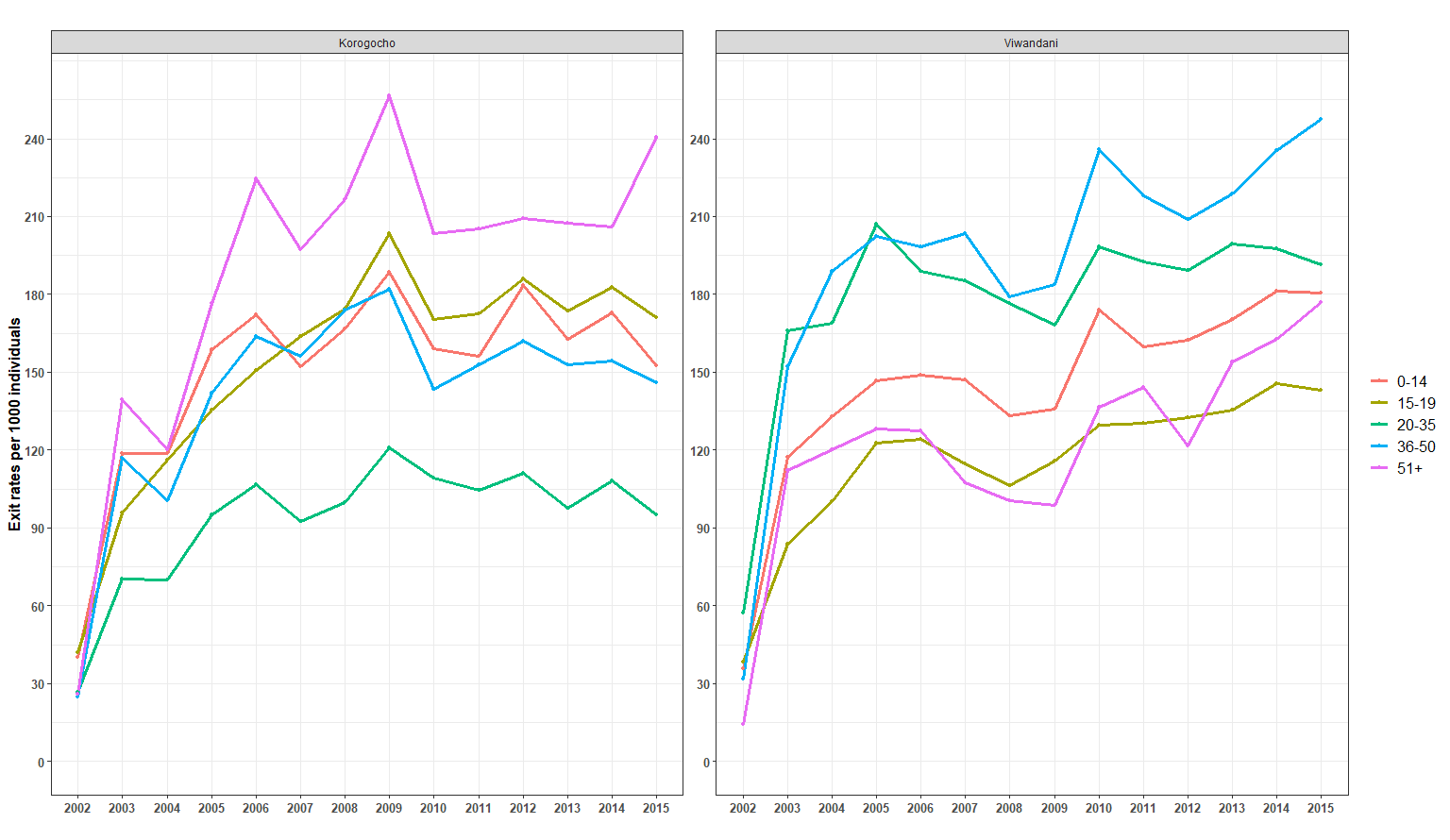


**Figure S1: Exit rates disaggregated by slum area**


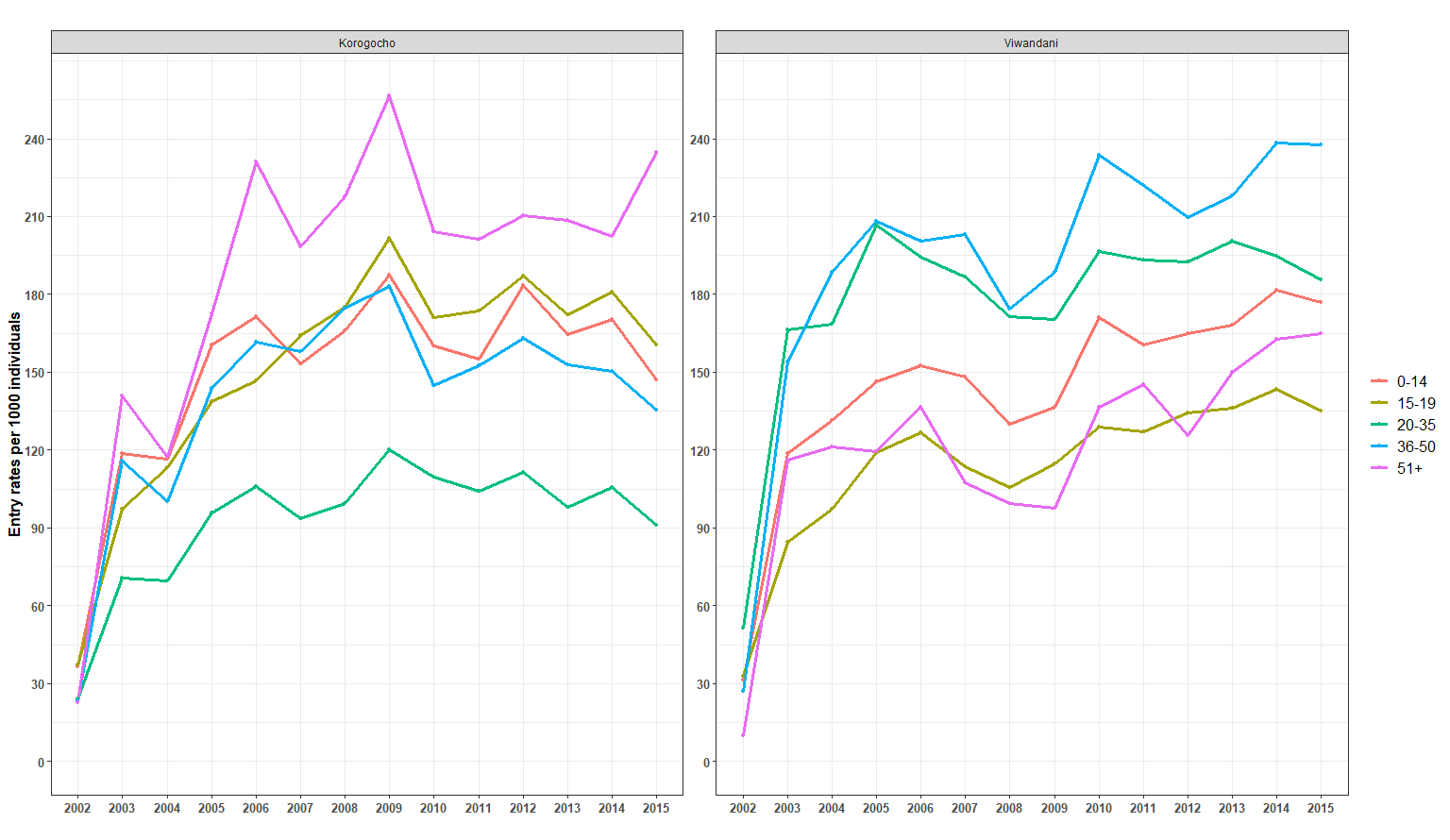


**Figure S1: Entry rates disaggregated by slum area**


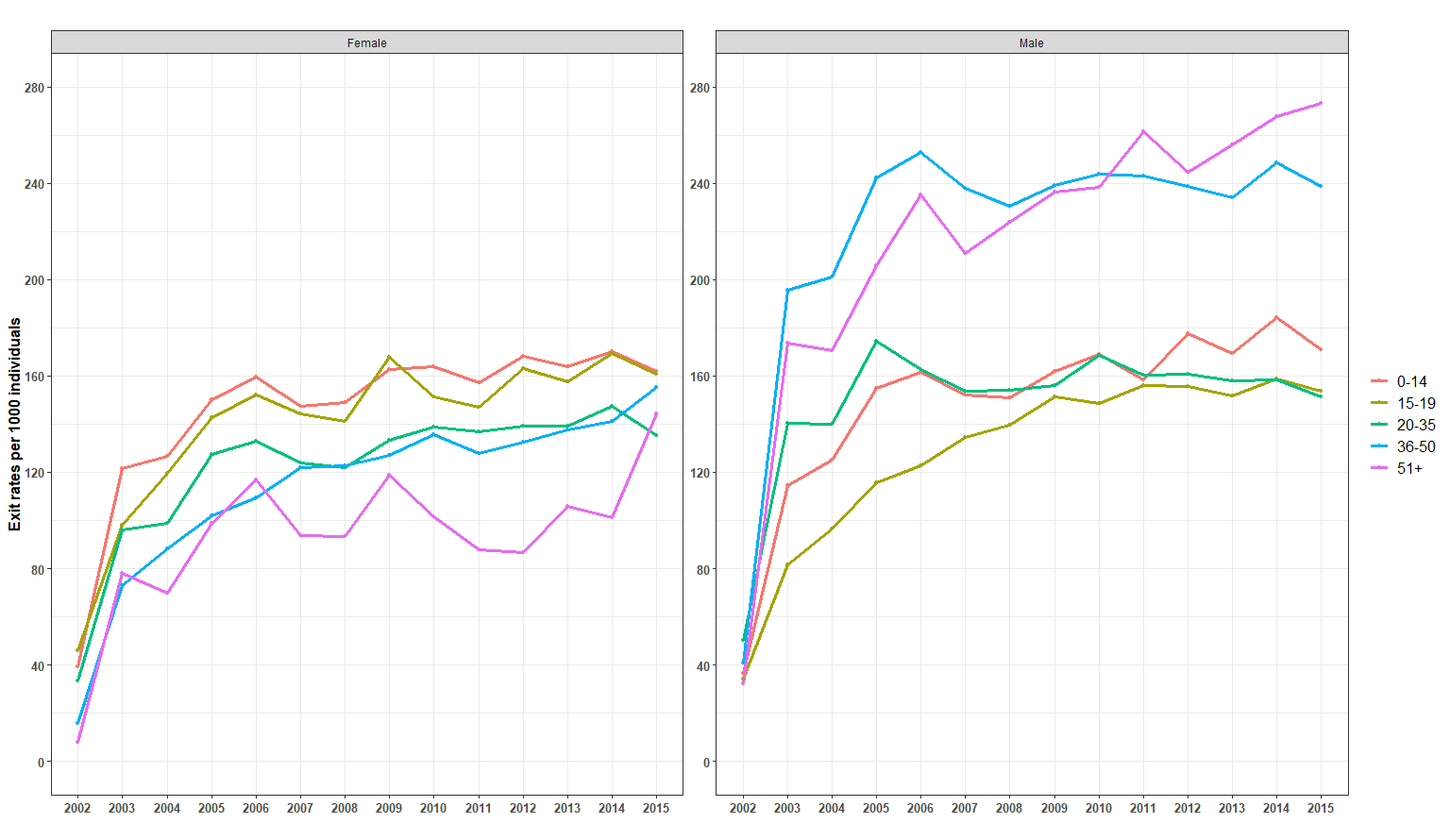


**Figure S3: Exit rates disaggregated by gender**


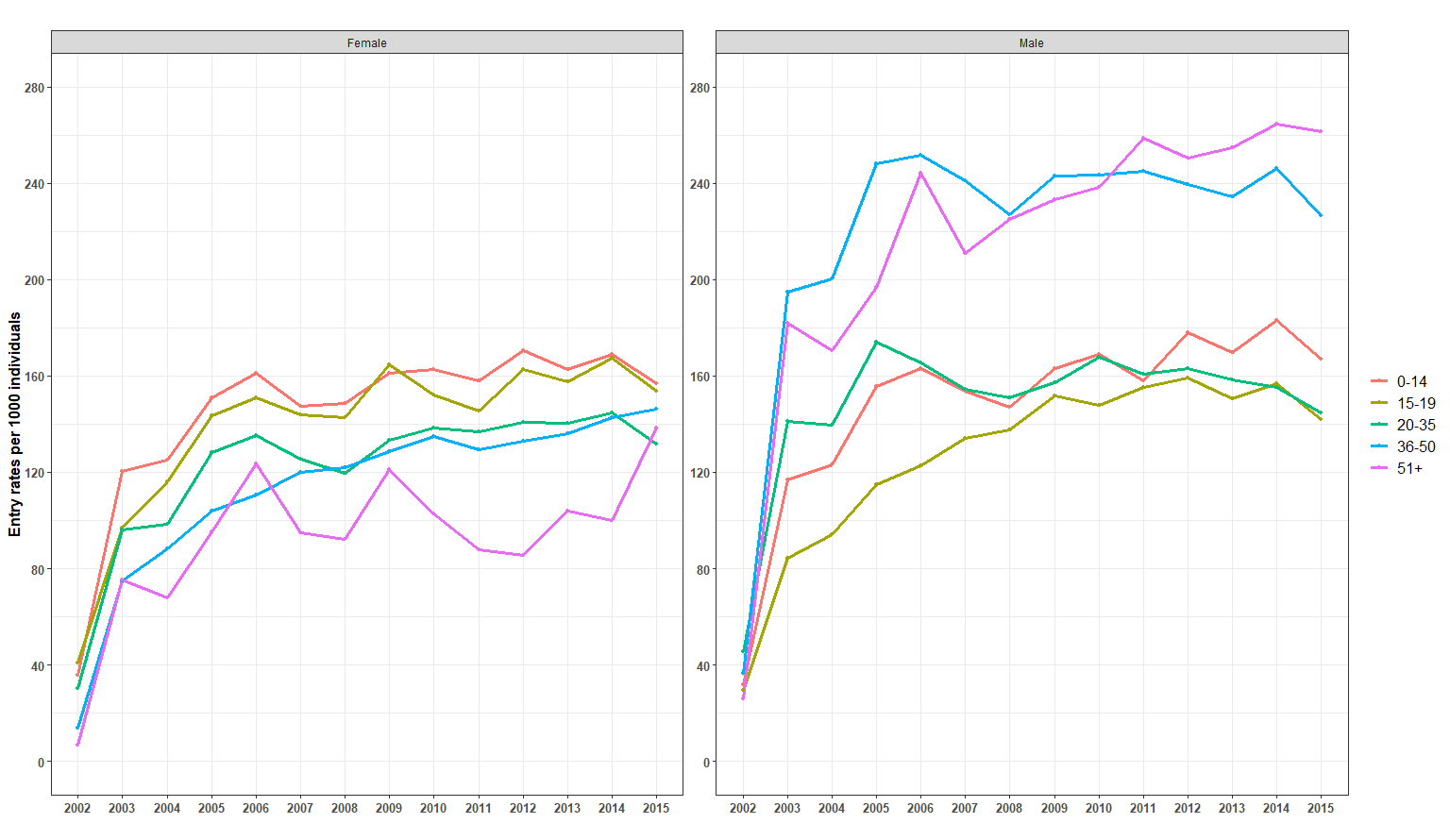


**Figure S4: Entry rates disaggregated by gender**
